# *Bacillus subtilis* isolated from medicinal plants rhizosphere effectively controls Cercospora leaf spot and improves plant growth in mung bean (*Vigna radiata*)

**DOI:** 10.3389/fmicb.2026.1850863

**Published:** 2026-08-19

**Authors:** Misbah ul Ain, Akhtar Hameed, Saima Muzammil, Tsanko Gechev, Muhammad Faisal

**Affiliations:** 1 Institute of Plant Breeding and Biotechnology (IPBB), MNS-University of Agriculture, Multan, Pakistan; 2 Institute of Plant Protection (IPP), MNS-University of Agriculture, Multan, Pakistan; 3 Institute of Microbiology, Government College University, Faisalabad, Pakistan; 4 Department of Molecular Stress Physiology, Center of Plant Systems Biology and Biotechnology, Plovdiv, Bulgaria; 5 Department of Molecular Biology, Plovdiv University, Plovdiv, Bulgaria

**Keywords:** *Bacillus subtilis*, *Cercospora canescens*, Cercospora leaf spot, eco-friendly management, foliar application, mung bean, seed priming

## Abstract

**Background:**

Mung bean is an important leguminous crop, which is reported to face devastating yield losses of up to 70% due to Cercospora leaf spot (CLS) disease. Traditional methods, such as the application of agrochemicals and fungicides, have been used to control CLS, but their intensive use has toxic effects on edible crops.

**Methods:**

To find out a sustainable alternative, this study characterizes a strain, *Bacillus subtilis* Medicinal_04, isolated from *Cannabis sativa* rhizosphere and explores its role as an eco-friendly biofungicide and biostimulant. The species level identification of the isolate was confirmed by Average Nucleotide Identity (ANIb) and a digital DNA–DNA hybridization (dDDH). The antagonistic efficacy of *B. subtilis* Medicinal_04 against *Cercospora canescens* was evaluated *in vitro* as well as *in planta* assays.

**Results:**

ANIb of 97.80% and a dDDH score of 85.90% against the reference *B. subtilis* str. 168. confirmed this isolate as *B. subtilis.* The *in-vitro* results showed that *B. subtilis* robustly inhibited *C. canescens* growth by 81.5%, strongly correlated with positive chitinolytic activity and a diverse genomic array of secondary metabolite biosynthetic gene clusters. The *in planta* results demonstrated that *B. subtilis* seed priming reduced disease incidence by 80 and 71.4%, while foliar application resulted in reductions of 90 and 85.7% for NM-51 and NM-20-21 varieties, respectively. Furthermore, fungicide application successfully reduced disease, however it caused noticeable phytotoxic reductions in root-shoot architecture and chlorophyll content. In contrast, biological interventions completely bypassed these trade-offs as *B. subtilis* application displayed improved root-shoot length, pod number, and chlorophyll content, while simultaneously enhancing antioxidative enzyme activities (SOD, POD, and CAT) and *PR-1* gene expression.

**Conclusion:**

These findings demonstrate that *B. subtilis* Medicinal_04 has the potential to serve as a multifunctional biocontrol agent and is capable of securing high-level disease suppression and optimizing plant productivity, offering a valuable toolkit for climate-smart, sustainable agriculture.

## 1 Introduction

Mung bean (*Vigna radiata* L.) is one of the pulses crops that belongs to the family Fabaceae (Zhao et al., 2022). It is mainly consumed as sprouts, dhal, and flour (Sakhare et al., 2026). Mung bean seeds are highly nutritious; 100 g of seed contains 59.7 g of carbohydrates, 24.3 g of protein, 6.1 g of crude fiber, 0.8 g of fats, and essential minerals such as potassium (1,285 mg), zinc (2.9 mg), iron (28.5 mg), and calcium (127 mg) (Abebe et al., 2024). Notably, mung beans protein exhibits several health benefits, including proven anti-cancer activities (Huang et al., 2024). Due to its health benefits, mung beans are increasingly utilized to develop functional foods, including nutritious bread, noodles, pasta, cookies, cake, biscuits, and healthy snacks (Sakhare et al., 2026). Furthermore, their techno-functional attributes, such as solubility and emulsification, enable versatile applications in the food industry to formulate antioxidant films, emulsion matrices, and meat analogues (Huang et al., 2024).

Global mung bean production stands at approximately 5.3 million tons, with 90% originating in Asia, primarily India, China, Myanmar, Pakistan, and Thailand (Australian Centre for International Agricultural Research, 2022). In the 2024–2025 cropping cycle, Pakistan produced approximately 131,000 tons (t) of mung beans from 185,000 hectares (ha), yielding a relatively low national average of 0.708 t/ha (Agriculture Marketing Information Services, 2025; Government of Pakistan, 2025). Recent estimates for the 2025–2026 season project a modest output increase to 150,800 tons (Federal Committee on Agriculture, 2026). However, domestic production consistently falls short of national requirements; this deficit necessitated massive imports during the FY2025 cycle, with total pulse imports exceeding 1.4 million tons and impacting foreign exchange reserves by roughly 1 billion USD (Pakistan Bureau of Statistics, 2026). In contrast, the global average for mung bean productivity is roughly 1.2 t/ha, with regional leaders like Myanmar achieving 1.42 t/ha (Myanmar Pulses, Beans, Maize and Sesame Seeds Merchants Association, 2025). Furthermore, experimental agronomic trials demonstrate a potential yield threshold of 2.05 t/ha through optimized crop management practices (Lakew, 2025). Consequently, the evaluation of the underlying stress factors responsible for this pronounced yield gap in Pakistan is imperative to formulate targeted strategies aimed at mitigating the country’s domestic production deficit.

Abiotic and biotic stresses were the major causes of the decline in mung bean production (Sharma et al., 2024). Key abiotic stresses in mung bean include drought, salinity, heat, and heavy metal toxicity (Sharma et al., 2024). Whereas biotic pressures comprise the mung bean yellow mosaic virus, dry root rot, anthracnose, Cercospora leaf spot, and insect pests such as aphids, jassids, and whiteflies (Ilyas et al., 2023; Sharma et al., 2024). Of stresses, biotic stress has been found to have more drastic effects and losses, especially the fungal diseases, which have resulted in 40 to 60% losses in Mung bean (Ajmal et al., 2024; Kumar et al., 2023).

Among these biotic threats, Cercospora leaf spot (CLS) stands out as one of the fungal diseases caused by a hemibiotrophic fungal pathogen in mung bean. The disease is generally spread by conidial spores that develop and mature on infected leaves, strongly correlated with high relative humidity and wind speed (Mahapatra et al., 2022). The specific causal agent of CLS in mung bean is *Cercospora canescens* Ellis and Martin, recognized as one of the most harmful soil- and air-borne fungi destroying mung bean cultivation in Pakistan (Abbas et al., 2020; Rafiq et al., 2022). The disease symptoms typically appear 30 to 40 days after sowing (DAS), depending on the availability of disease-favorable conditions in fields, and are characterized by circular/irregular reddish-brown edges, yellow halos, and dark necrotic lesions that rapidly enlarge during the critical flowering and pod-filling stages (Ilyas et al., 2023; Sahoo et al., 2022). Severe infestations have often been reported to result in complete defoliation and poor pod development, and an overall reduction in seed yield per plant (Sahoo et al., 2022). Under natural epiphytotic environments in Pakistan, the pathogen compromises both seed quality and foliage, causing severe quantitative yield losses that typically range from 50 to 70% (Rafiq et al., 2022) and escalate up to 95–96% in highly susceptible local cultivars under optimal disease-conducive conditions (Abbas et al., 2020; Ilyas et al., 2023).

While various chemical treatments exist for controlling plant fungal diseases, their efficacy is severally constrained and limited for consumable crops due to rapid resistance build-up in pathogens and critical environmental risks (El-Saadony et al., 2022). In the intensive agricultural systems of Pakistan, the persistent overuse of agrochemicals, especially synthetic fungicides, triggers acute and chronic human illnesses, while disrupting agro-ecosystems by eliminating non-target beneficial organisms and contaminating surrounding aquatic life (Ali et al., 2022; Said, 2023). Specifically, heavy fungicides applications drive a sharp decline in soil microbial biomass and diversity, suppressing functional and beneficial rhizobacteria which are vital for essential nutrient cycling, carbon stabilization, and nitrogen fixation (Nagrale et al., 2023; Zhang et al., 2024). This increasing biological degradation fundamentally undermines natural soil fertility across local cultivation zones, posing a long-term threat to local as well as global food security and environmental health (Zhang et al., 2024). Due to these compounding environmental and biological degradation problems, the global agricultural sector is moving toward sustainable, biopesticide or biofungicide-based management strategies that safeguard public health while protecting soil fertility and environmental integrity. These bio-fungicides are primarily derived from beneficial microbial antagonists, botanical extracts, and natural biochemicals that offer a highly targeted approach to crop protection.

In particular, biological control exploiting plant growth-promoting rhizobacteria (PGPR) offers a sustainable, eco-friendly, and efficient defense mechanism to replace chemicals. Indigenous PGPR strains shield host plants against destructive pathogens through highly coordinated mechanisms, including the production of localized antibiotics, antimicrobial peptides, bacteriocins, volatile metabolites, and lytic cell wall-degrading enzymes (Dhanabalan et al., 2024). *Bacillus* spp. represents a standout bacterial genus among PGPR, thriving within competitive rhizospheric environments by producing a synergistic array of secondary metabolites. Specifically, *Bacillus subtilis* has been deployed to control destructive pathogens like *Fusarium oxysporum* and *Rhizoctonia solani,* while concomitantly promoting plant growth and development (Cao et al., 2011; Yang et al., 2024). This genus employs multi-tiered direct and indirect defense mechanisms by establishing competitive niche exclusion, producing secondary metabolites, cell wall-degrading enzymes, and activating induced systemic resistance (ISR) pathways within the host plant (Franco-Sierra et al., 2020; Hashem et al., 2019; Yang et al., 2024). This biological priming not only enhances overall plant growth and crop yield but also strengthens the immune response(s) through the controlled accumulation of reactive oxygen species (ROS), elevated activation of key defense-related genes (PRs) and plant defensin (PDFs), as well as the enhanced expression of antioxidative enzymes (such as peroxidase and catalase) (Siddika et al., 2024; Yadav et al., 2024).

Recent literature demonstrates that *Bacillus subtilis* strains isolated from the rhizosphere of medicinal plants exhibited potent biocontrol activity against devastating plant fungal pathogens. Specifically, rhizospheric strains isolated from garlic (*Allium sativum*) (Faquin et al., 2025), mangrove (*Rhizophora mangle*) (Shinde et al., 2025), cannabis (*Cannabis sativa* L.) (Corredor-Perilla et al., 2023), wild mint (*Mentha haplocalyx* Briq.) (Li et al., 2023), and turmeric (*Curcuma longa* L.) (Chenniappan et al., 2019) have shown significant antagonistic efficacy against fungal pathogens such as *Stromatinia cepivora, Aspergillus niger, Rhizopus arrhizus, Mucor mucedo, Fusarium* spp., and specialized turmeric rhizome rot pathogens, respectively (Chenniappan et al., 2019; Faquin et al., 2025; Li et al., 2023; Shinde et al., 2025). These specialized antagonistic bacteria protect host plants through diverse direct and indirect mechanisms, including the secretion of antifungal lipopeptides, the production of cell-wall-degrading enzymes (cellulase, amylase, chitinase, and *β*-1,3-glucanase), the synthesis of antibiotics (2,4-diacetylphloroglucinol, pyrrolnitrin, pyoluteorin, bacillomycin D, fengycin, hydrogen cyanide), antimicrobial compounds (palmitic acid derivatives, fatty acid amides, and pyridine-based molecules) and the induction of systemic plant resistance (ISR) (Chenniappan et al., 2019; Corredor-Perilla et al., 2023; Li et al., 2023; Shinde et al., 2025).

*Cannabis sativa* has a long history of recreational, medicinal, and religious use, displaying deep therapeutic potential in modern medicine (Malabadi et al., 2024). Agronomically, the crop is robust and generally pest-free, though it faces flea beetle issues (Bano et al., 2022). *Cannabis* plant dense canopy suppresses weed growth and development, allowing it to compete effectively in the field (Bano et al., 2022). Notably, the Cannabis rhizosphere harbors beneficial bacterial communities including *Bacillus* spp., *B. oceanisediminis, B. licheniformis*, *B. zhangzhouensis, B. subtilis* strains, *Priestia aryabhattai*, *Bacillus* sp.*, Serratia marcescens,* and *Stenotrophomonas maltophilia* strains; and *B. subtilis* strains; these microflora have been shown to suppress *Fusarium* spp. (Corredor-Perilla et al., 2023) and actively enhance plant growth parameters (Bano et al., 2022).

Therefore, the current study aims to isolate microbe(s) such as *Bacillus subtilis* strain(s) from the *Cannabis sativa* rhizosphere to be highly effective as biocontrol and plant development and systematically investigate their *in vitro* antifungal efficacy against Cercospora leaf spot (CLS) disease. Furthermore, this study evaluates the strain’s downstream effects on the morphological, physiological, and biochemical parameters, as well as the expression of the *PR-1* defense gene, in mung beans (*Vigna radiata*) subjected to CLS stress using both seed priming and foliar application methodologies.

## 2 Materials and methods

### 2.1 Isolation, phenotypic profiling, biochemical and plant growth-promoting rhizobacteria (PGPR) characterization of *Bacillus subtilis* Medicinal_04

The biocontrol agent *Bacillus subtilis* Medicinal_04 was isolated from the rhizospheric soil of *Cannabis sativa* L. plant from Lahore, Pakistan. The rhizospheric soil samples were collected from 5 distinct and different sites at depths of 10–15 cm and thoroughly blended in sterile bags to ensure a completely homogeneous composite mixture. A total of seven isolates were obtained, purified and submitted in NCBI under PRJNA1290071 project, and one of these isolates was identified as *B. subtilis,* the details of which can be found from https://www.ncbi.nlm.nih.gov/bioproject/1290071. The *B. subtilis* strain was further purified from soil samples using a standard serial dilution plating method followed by the selection of single colonies using the quadrant streaking technique on Luria-Bertani (LB) agar plates as described by Masi et al. (2021). For long-term preservation, the purified *B. subtilis* Medicinal_04 was grown in LB broth overnight at 25 °C and stored in a 60% (v/v) glycerol solution at −80 °C for future investigations. The purified isolate was initially classified using Gram staining and macro-morphologically characterized by size, shape, margin, elevation, and pigmentation. The definitive physiological and biochemical identification was performed using a number of standard diagnostic assays such as IMViC (Indole, Methyl Red, Voges-Proskauer, and Citrate utilization), catalase, urease, oxidase, mannitol fermentation, nitrate reduction, and SIM (sulfur, indole, motility) tests with three replicates.

The plant growth-promoting rhizobacteria (PGPR) traits of *B. subtilis* Medicinal_04 were characterized using a suite of standard *in vitro* assays. The quantitative estimation of Indole-3-acetic acid (IAA) production was measured calorimetrically at 530 nm using Salkowski’s reagent following a 48-h incubation in LB broth supplemented with 0.1% L-tryptophan, as described by Ayaz et al. (2024). The siderophore production was assessed on chromeazurol-S (CAS) agar medium according to the protocol developed by Patel et al. (2023). The isolate was inoculated onto Pikovskaya’s agar for inorganic phosphate solubilization qualitative evaluation, and positive activity was identified by the formation of distinct, clear halo zones around bacterial colonies after 5 days of incubation at 30 °C (Gupta et al., 2022). Ammonia production was detected by adding Nessler’s reagent to 72-h-old peptone water cultures, where a distinct color transition to yellow-orange confirmed a positive reaction (Patel et al., 2023). Additionally, catalase activity was verified by observing the immediate liberation of oxygen bubbles upon exposing fresh colony biomass to a 3% hydrogen peroxide solution, while chitinolytic activity was determined on agar media amended with colloidal chitin, as described by Baard et al. (2023). All PGP assays were performed in triplicate.

### 2.2 Genomic and *in silico* characterization of *Bacillus subtilis* Medicinal_04

Molecular identification of *B. subtilis* Medicinal_04 was performed following protocols adapted from Rochelle et al. (1992). The strain was cultured in LB medium (30 °C, 24 h, 120 rpm), and genomic DNA was extracted using the Qiagen DNeasy Mini Kit, quantified via NanoDrop, and verified on a 0.70% agarose gel. The 16S rRNA gene was amplified using universal primers 8F (5′AGTTGATCCTGGCTCAG3′) and reverse primer 1492R (5′TACCTTGTTACGACTT3′), followed by sequencing and species identification. The whole genome sequencing (WGS) of the same DNA was performed by MicrobesNG, Birmingham, UK,^1^ kindly supported by GetGenome, Norwich, UK,^2^ to achieve high-resolution taxonomic placement. Genomic DNA libraries were prepared using the Nextera XT Library Prep Kit (Illumina, San Diego, United States) and sequenced on Illumina NovaSeq 6000 (Illumina, San Diego, United States) using a 250 bp paired-end protocol. Reads were adapters trimmed using Trimmomatic version 0.30 (Bolger et al., 2014), assembled via SPAdes version 3.7, and annotated using Prokka 1.11 (Seemann, 2014). Sequence alignment against the GenBank database demonstrated >99% homology, confirming the strain’s identity as *B. subtilis* Medicinal_04. The sequence data were deposited in the National Center for Biotechnology Information (NCBI) database under SRA accession SRS25760486, BioSample SAMN49909479, and Genome Assembly GCA_056674935.1.

Since 16S rRNA gene sequencing assigned this isolate as *Bacillus subtilis,* however, the precise taxonomic identification of *B. subtilis* Medicinal_04 was further validated by two gold-standard methods: digital DNA–DNA hybridization (dDDH) and average nucleotide identity (ANI). The *B. subtilis* Medicinal_04 genome was compared against reference genomes of closely related *Bacillus* species. The dDDH similarity values were calculated using the Genome-to-Genome Distance Calculator version 3.0 (GGDC; https://ggdc.dsmz.de/) (Fessia et al., 2024) using default settings. A standard dDDH threshold of ≥ 70% was applied for species-level delineation. Concurrently, average nucleotide identity (ANI) values based on BLAST (ANIb) were determined using the JSpeciesWS web server^3^ (Martinez-Vidales et al., 2024). The standard threshold of 95% ANIb was applied as the species boundary, where values exceeding this baseline (above 95%) indicated that the strains belonged to the same species (Martinez-Vidales et al., 2024).

The BLAST Ring Image Generator (BRIG) was exploited to simultaneously compare a newly sequenced strain against multiple reference genomes (Sohail et al., 2025). BRIG generates a circular map wherein the query genome serves as the central anchor, around which concentric rings representing reference genome(s) are concentrically overlaid. Gaps within these reference rings visually delineate regions of DNA that are unique to the query strain, which often harbor prophages, genomic islands, or specialized virulence/fitness factors. Thus, the whole-genome assembly of *B. subtilis* Medicinal_04 (accession: JBWWQS000000000) was compared against three reference *Bacillus* strains: *B. subtilis* subsp. *subtilis* str. 168 (accession no. NC_000964.3), *B. velezensis* strain NKYL29 (accession no. NZ_JPYY00000000.1), and *B. amyloliquefaciens* strain SRCM123386 (accession no. CP128501.1) using BRIG with default settings.

Members of the genus *Bacillus* are well-documented and known for producing diverse antimicrobial lipopeptides (surfactin, iturin, fengycin) and polyketides (Dhanabalan et al., 2024). In contrast, reference strain 168 is highly domesticated, having lost or muted several of these metabolic capabilities through prolonged laboratory cultivation (Zeigler et al., 2008). To systematically evaluate the functional biocontrol and metabolic potential of *B. subtilis* Medicinal_04, its whole genome sequence was mined. Secondary metabolite biosynthetic gene clusters (BGCs) were predicted using antiSMASH 8.4.0 under default parameter settings (Blin et al., 2025). Preliminary NCBI BLAST profiling demonstrated high sequence similarity between the query strain and the reference strain *B. subtilis* subsp. subtilis str. 168 within shared core genomic regions.

### 2.3 Standardisation and preparation of fungal and bacterial inocula

The *Cercospora canescens* Ellis and Martin (pathogen) culture was obtained from Ayub Agriculture Research Institute (AARI), Faisalabad, and sub-cultured on potato dextrose agar (PDA) media to freshen the inoculum. For subsequent bioassay, the *C. canescens* conidial spore suspension was harvested from a 7-day-old culture plate and spore concentration (5 × 10^5^ spores/mL) was adjusted using sterile distilled water and Neubauer hemocytometer as described by Babar et al. (2022). This specific fungal concentration of 5 × 10^5^ spores/mL was selected because it represents a well-established baseline optimized across historical regional studies (Abbas et al., 2020; Shahbaz et al., 2014) to ensure successful, reproducible disease manifestation under controlled conditions without causing excessive, non-target tissue necrosis. The pathogenicity of *C. canescens* isolate was confirmed by Koch’s postulates as described by Rafiq et al. (2022). Concurrently, the isolated *B. subtilis* Medicinal_04 strain was sub-cultured by streaking onto Luria Bertani (LB) agar media. To prepare the antagonist treatment, the bacterial culture was diluted with sterile distilled water by serial dilution method to a consistent and final working density of 1 × 10^6^ CFU/mL prior to application (Akbar et al., 2019; Maslennikova et al., 2023). These operational thresholds were maintained as constant parameters based on established literature to strictly prioritize the comparative efficacy of the application pathways (seed priming versus foliar spray).

### 2.4 Preparation and standardization of the chemical fungicide control

A commercial fungicide, containing a formulation of 4% Metalaxyl-M and 64% Mancozeb and commonly utilized in localized mung bean cultivation to manage Cercospora leaf spot (Shahbaz et al., 2014) was procured from a local market. Fungicide was used as a positive control to compare against the biocontrol efficacy of *B. subtilis* Medicinal_04 strain. A standardized chemical stock solution was prepared by dissolving 300 mg of the fungicide in 100 mL of sterile distilled water to achieve a working concentration of 3,000 ppm as described by Rajput et al. (2012).

### 2.5 *In vitro* antagonistic bioassay of *Bacillus subtilis* Medicinal_04

The antagonistic efficacy of *B. subtilis* Medicinal_04 against *C. canescens* was evaluated using the disc-diffusion method with minor modifications adapted from Hidayat et al. (2023). Briefly, sterile filter paper discs (5 mm diameter) were immersed for 2 min in either the *B. subtilis* suspension (1 × 10^6^ CFU/mL) or the standardized fungicide solution (300 mg/100 mL of water). Subsequently, four treated discs were equidistantly placed on each PDA petri plate pre-inoculated with *C. canescens*. The PDA plate containing *C. canescens* alone served as a negative control. All petri plates were incubated at 28 °C for seven days, after which zones of inhibition were measured using Image J software. The experiment was conducted in triplicate. The diameter of fungal growth inhibition was calculated according to the formula (Terefe et al., 2023):


$$\text{Inhibition}\;(\%)=\frac{\mathrm{DC}-\mathrm{DT}}{\mathrm{DC}}\times 100$$

Where,

DC is the average diameter of linear growth in the control.

DT is the average diameter of linear growth in treatment.

### 2.6 In planta biocontrol evaluation: plant material, soil conditions, and experimental layout

Mung bean seed varieties, NM-51 (comparatively CLS-resistant) and NM-20-21 (CLS-susceptible), were obtained from the Nuclear Institute for Agriculture and Biology (NIAB), Faisalabad. The seeds were surface sterilized with sodium hypochlorite (NaClO) solution for 3 min and subsequently rinsed multiple times with distilled water as described by Choudhary and Chand (2022). For the biological treatment group, the sterilized seeds were immersed for 4 h in bacterial suspension (1 × 10^6^ CFU/mL) prepared in 1% carboxymethyl cellulose (CMC) carrier solution, while the control group seeds were concurrently soaked in sterile distilled water as described by El-Mohamedy and Alla (2013).

The trial was conducted within a specialized lath house facility under natural daylight, maintaining an approximate 12 h light/12 h dark photoperiod. Over the 60-day experimental timeframe, the ambient temperature ranged between 27 °C to 32 °C. Relative humidity (75–85%) was maintained through structured, routine watering and intermittent mist spraying inside the facility to keep favorable conditions for *C. canescens* pathogenesis. The CLS-challenged plants were placed in a physically isolated zone of the lath house away from the mock-treated control plants.

Pots were filled with a pre-autoclaved substrate mixture of soil, sand, and decomposed cattle manure in a 2:1:1 (w/w/w) ratio, with adequate moisture conditions maintained to support optimal germination and vegetative growth. Physicochemical analysis was performed, which showed that the experimental soil mixture possessed a pH of 7.45, an electrical conductivity (EC) of 0.68 dS m^−1^, and a sandy loam texture. The nutritional profile of the matrix consisted of 0.82% organic carbon, 0.31% total nitrogen, 48 kg/ha available phosphorus, and 286 kg/ha exchangeable potassium. Soil micronutrient concentrations comprised calcium (1720 kg/ha), zinc (1.89 ppm), manganese (25.8 ppm), copper (10.2 ppm), and iron (30.4 ppm). The treatment applications were administered in a strict chronological sequence. This soil mixture is highly favorable for plant growth and interaction with PGPRs, as its neutral pH, low salinity, and organic-rich sandy loam texture provide ideal conditions for root colonization and PGPR activity.

The experiment was arranged in a completely randomized design (CRD) under a factorial layout with three independent biological replicates (pots) per treatment and five plants per pot. To avoid pseudoreplication and artifacts of inflated freedom and error type 1, the data for all parameters were measured for all five plants within each pot individually and then averaged to generate a single true value for each independent experimental unit (biological replicate, *n* = 3) (Samet, 2026; Ziotti et al., 2026).

Foliar application of both antagonist suspensions (*B. subtilis* 1 × 10^6^ CFU/mL) and commercial chemical fungicide (300 mg/100 mL) was applied on plant leaves 19 days after sowing (DAS) using separate manual atomizing sprayers to prevent cross-contamination. The plants from all groups were then challenged with *C. canescens* suspension (5 × 10^5^ spores/mL) 21 DAS by the foliar application as described by Babar et al. (2022). The plants were harvested at 60 DAS to evaluate growth parameters. The comprehensive framework detailing the individual *in planta* treatment combinations is summarized in Table 1.

**Table 1:** Acronyms and treatments details.

Treatment code	Priming/application type	Treatment/inoculation
HP	Hydro-primed (negative control)	No
HP + CC	Hydro-primed *+* Foliar-application of *C. canescens* (positive control)	*C. canescens* (5 × 10^5^ spores/mL)
HP + F	Hydro-primed *+* Foliar-application of Fungicide	Fungicide (300 mg/100 ml of water)
HP + F + CC	Fungicide *+* Foliar-application of *C. canescens*	Fungicide (300 mg/100 ml of water) + *C. canescens* (5 × 10^5^ spores/mL)
BS(P)	*B. subtilis*-primed	*B. subtilis* (1 × 10^6^ CFU/mL)
HP + BS(F)	Hydro-primed *+ B. subtilis*-foliar-application	*B. subtilis* (1 × 10^6^ CFU/mL)
BS(P) + CC	*B. subtilis*-primed + Foliar-application of *C. canescens*	*B. subtilis* (1 × 10^6^ CFU/mL) + *C. canescens* (5 × 10^5^ spores/mL)
HP + BS(F) + CC	Hydro-primed *+ B. subtilis*-foliar-application + Foliar-application of *C. canescens*	*B. subtilis* (1 × 10^6^ CFU/mL) + *C. canescens* (5 × 10^5^ spores/mL)

### 2.7 Disease incidence (DI) measurements

Plants were classified as infected when at least one characteristic CLS lesion was observed on its leaves however all included leaves were observed to have more 2 lesions. Initial disease symptoms began to appear around 48 h after inoculation (hai); however the disease incidence (DI) was recorded and evaluated 30 DAS or 9 days after inoculation (DAI) to allow symptoms to be fully and uniformly developed across all plants. DI was determined according to the methodology described by Awan and Shoaib (2019) and Soliman et al. (2023).


$$\mathrm{DI}\%=\frac{\text{Number infected plant}\;}{\text{Total number of plants}}\times 100$$

The disease reduction percentage (DRP) over positive control was measured by following formula as described by Cao et al. (2011).


$$\mathrm{DRP}=1-\frac{\;\mathrm{DT}}{\mathrm{DC}}\times 100$$

Where DC and DT are the disease incidence percentages in control (positive control) and treatments, respectively.

### 2.8 Measurement of morphological, physiological traits and biochemical parameters

Shoot length (cm), root length (cm), and the number of pods of plants from all treatments were measured 60 DAS. The measurements were averaged (five plants within each pot) to generate a single value representing one biological replicate (pot), which was considered the independent experimental unit because all plants within a pot shared the same soil and growing conditions. Chlorophyll contents were measured (30 DAS) from the third trifoliate leaves by Chlorophyll Meter (502-SPAD Spectrum, Konica Minolta, Japan).

For biochemical analysis, fully mature first trifoliate leaf samples were collected from all mung bean treatments on 24 DAS and 72 h after inoculation (hai) with *C. canescens* and immediately stored at −80 °C. Leaf samples (1 g) from all treatments of mung bean plants were crushed in a mortar and pestle with 5.0 mL of 0.1 M phosphate buffer and centrifuged at 10,000 rpm at 4 °C. The obtained homogenate supernatant (crude enzyme extract) was shifted to new tubes and used to estimate downstream SOD, POD, and CAT activities (Choudhary and Chand, 2022).

For the SOD measurement, each sample was prepared by adding 20 μL methionine, 20 μL Triton X-100, 10 μL NBT, 10 μL crude enzyme extract, 50 μL potassium phosphate buffer (pH 7.0), 80 μL distilled water, and 10 μL riboflavin to make a total of 200 μL reaction. This mixture was loaded into UV-transparent 96-well plates and subsequently exposed to white fluorescent light (Approx. 4,000 lux) for 20 min to initiate the reaction. The absorbance of SOD was measured at 560 nm using an ELISA reader as described by Giannopolitis and Ries (1977) and Mahmood et al. (2022).

For the POD measurement, the reaction mixture consisted of 160 μL potassium phosphate buffer, 20 μL H2O2 (40 mM), 20 μL guaiacol (20 mM), and 20 μL crude enzyme extract to reach a total volume of 220 μL. The prepared samples were loaded into UV-transparent 96-well plates for spectrophotometric analysis via an ELISA reader equipped for kinetic analysis, and the increase in absorbance of POD was measured at 470 nm for 3 min to determine the change in absorbance per minute (ΔA/min) as described by Chance and Maehly (1955) and Mahmood et al. (2022).

For the CAT measurement, the reaction mixture was prepared by mixing 50 μL crude enzyme extract, 90 μL distilled water, 40 μL potassium phosphate buffer (pH 7.0), and 20 μL H_2_O_2_ (15 mM) to reach a total volume of 220 μL. The prepared samples were loaded into UV-transparent 96-well plates for spectrophotometric analysis via an ELISA reader equipped for kinetic analysis, and the decrease in absorbance of CAT was measured at 240 nm for 3 min to determine the ΔA/min as described by Chance and Maehly (1955) and Mahmood et al. (2022).

The activity of each enzyme was normalized and compared with Bovine Serum Albumin (BSA) as a standard using the Bradford assay technique. Briefly, the total soluble protein concentration of the crude extract was determined by comparing its absorbance against BSA serial dilutions and standard curve. Finally, the activities of SOD, POD, and CAT were normalized by dividing the enzyme activity units by the total protein content, expressing the results as Specific Activity in Units per milligram of protein (U/mg protein).

### 2.9 RNA extraction, cDNA synthesis, and gene expression analysis

Fully expanded first trifoliate leaves were collected 72 hai (24 DAS) from all treatments, immediately snap-frozen in liquid nitrogen, and packed into zipper bags. All samples were then transferred to −80 °C for long-term storage. Total RNA was extracted from mung bean leaves using TRIzol reagent (Invitrogen), according to the manufacturer’s instructions. Subsequently, cDNA was synthesized from 0.5 ug of total RNA using a Thermo Fisher kit. The nucleotide sequence of the *PR-1* gene (Gene ID 106769349, accession number NC_028357.1) was retrieved from NCBI,^4^ and primers were designed using PerlPrimer v1.1.21^5^ (Supplementary Table S1). The *UBQ* gene (accession number, XM_014636394.1) was selected as the housekeeping gene, and the primer sequence was obtained from a previous study (Ke et al., 2021). Real-time qRT-PCR was performed, and relative gene expression of *PR-1* was quantified using 2^-ΔΔCT^ method as described by Livak and Schmittgen (2001) and Faisal et al. (2020).

### 2.10 Statistical analysis

All data are presented as means and standard errors (SEM). Microsoft Excel was used for data organization and calculation of descriptive statistics, while statistical analyses were performed using GraphPad Prism.^6^ Statistical analyses were conducted using the mean values of the three independent biological replicates (pots). One-way ANOVA followed by Tukey’s multiple comparison test was used to compare a single experimental factor (i.e., comparison among treatment groups). Two-way ANOVA followed by Tukey’s multiple comparison test was used to evaluate the effects of two factors, namely treatment and mung bean variety (NM-51 and NM-20-21). In statistical significance testing, “ns” denotes a non-significant (*p* > 0.05), where asterisks (*) indicate increasing levels of statistical significance, with the *p* value of equal to or less than 0.05 (*p* ≤ 0.05).

## 3 Results

### 3.1 Isolation and characterization of the *Bacillus subtilis* Medicinal_04

Previous studies (Faquin et al., 2025; Shinde et al., 2025) have highlighted that *B. subtilis* strains isolated from the rhizosphere of medicinal plants exhibited biocontrol activity against destructive plant fungal pathogens. Therefore, the current study aimed to isolate and characterize a native antagonistic bacterium from *Cannabis sativa* L. rhizospheres. The results show that the *B. subtilis* strain was successfully isolated, purified, and taxonomically identified by comprehensive morpho-biochemical profiling. The results of morphological and biochemical studies are summarized in Supplementary Table S2. The morphological characterization confirmed that the isolate, designated as *Bacillus subtilis* Medicinal_04, is a gram-positive, motile, endospore-forming, rod-shaped bacterium (Bacilli). Biochemical evaluation revealed that the isolate was positive for citrate utilization, Voges-Proskauer (VP) reaction, mannitol fermentation, starch hydrolysis, nitrate reduction, and casein hydrolysis. Conversely, the strain was tested negative for indole production, methyl red (MR), oxidase activity, and gas production from glucose fermentation. Based on phenotypic and biochemical profiling, the rhizospheric isolate was identified and confirmed as *Bacillus subtilis* and assigned the nomenclature *Bacillus subtilis* Medicinal_04.

Members of the genus *Bacillus* have been known for exhibiting vital PGPR traits such as ammonia production, catalase activity, prominent chitinolytic activity, indole-3-acetic acid (IAA) production, and inorganic phosphate solubilization (Patel et al., 2023). In this study, the functional screening of the rhizospheric isolate *B. subtilis* Medicinal_04 revealed robust and versatile PGPR characteristics (Supplementary Table S2). The morpho-biochemical and physiological profiling demonstrated that the strain possesses strong plant growth-promoting potential. Specifically, the isolate exhibited highly prominent chitinolytic activity (++) alongside robust ammonia production (+) and strong catalase activity (+). Furthermore, qualitative testing confirmed that the strain was positive for indole-3-acetic acid (IAA) production (+). Conversely, under the tested *in vitro* conditions, the strain was negative for inorganic phosphate solubilization (−). Collectively, these phenotypic findings demonstrate that *B. subtilis* Medicinal_04 possesses substantial PGPR potential, may be attributed to its strong capacity for ammonia generation, catalase and chitinase expression, and inducible IAA synthesis.

### 3.2 Genomic characterization of *Bacillus subtilis* Medicinal_04

Since phenotypic traits and exhibition of PGP traits are insufficient evidences for definitive species-level resolution, a multi-tiered molecular and phylogenomic approach was deployed. Initial genus-level taxonomy of the isolate was resolved via 16S rRNA gene sequencing. A subsequent NCBI BLASTn search showed 99% identity with *Bacillus subtilis* strains, successfully establishing its generic placement. To achieve high-resolution taxonomic placement and secure solid species-level validation, whole-genome sequencing (WGS) of the *B. subtilis* Medicinal_04 genome was performed. The generated whole-genome assembly (accession: JBWWQS000000000) was subjected to rigorous, gold-standard phylogenomic analyses against closely related reference strains within the *Bacillus* genus, specifically *Bacillus subtilis* subsp. *subtilis* str. 168, *Bacillus velezensis* strain NKYL29, and *Bacillus amyloliquefaciens* strain SRCM123386. The pairwise Average Nucleotide Identity based on NCBI (ANIb) values calculated via JSpeciesWS revealed that *B. subtilis* Medicinal_04 shared 97.80, 75.34, and 76.73% sequence similarity with *B. subtilis* str. 168, *B. velezensis* NKYL29, and *B. amyloliquefaciens* SRCM123386, respectively (Table 2). Given that the established boundary for species-level delineation is >95 ANIb, the high (97.80%) similarity confirmed that the query isolate belongs to the same species as *B. subtilis* str. 168, whereas the low identity values (76.73%) marked this isolate as a distinct species from *B. velezensis* and *B. amyloliquefaciens*.

**Table 2:** Genome-based taxonomic identification of *B. subtilis* Medicinal 04 (accession no: JBWWSQ000000000) using ANI and dDDH analyses.

Query genome	Reference genome strain	ANIb (%)	dDDH	Probability of dDDH = 70%	G + C difference (%)	Status
JBWWSQ000000000	*B. subtilis* (NC_000964.3)	97.80%	85.90%	94.17%	0.10%	Same Species
JBWWSQ000000000	*B. velezensis* (NZ_JPYY00000000.1)	75.34%	20.70%	0.00%	2.17%	Distinct Species
JBWWSQ000000000	*B. amyloliquefaciens* (CP128501.1)	76.73%	21.00%	0.00%	1.84%	Distinct Species

This taxonomic placement was further validated by digital DNA–DNA hybridization (dDDH) using Genome-to-Genome Distance Calculator (GGDC) analysis. The dDDH values between *B. subtilis* Medicinal_04 and the reference strains for *B. subtilis*, *B. velezensis*, and *B. amyloliquefaciens* were determined to be 85.90, 20.70, and 21.00%, respectively (Table 2). These results strongly support the ANIb findings; the values for *B. velezensis* and *B. amyloliquefaciens* were significantly below the universally accepted 70% dDDH species-cutoff threshold, with a 0.00% probability of dDDH 70%. Conversely, the dDDH value with *B. subtilis* str. 168 (85.90%) successfully exceeded the species boundary line with a 94.17% probability of dDDH 70%, while exhibiting a minimal G + C genomic difference of only 0.10%. Taken together, these congruent phylogenomic datasets conclusively validate the identity of the rhizospheric isolate as *B. subtilis*, designating it as *B. subtilis* Medicinal_04.

**Figure 1:**
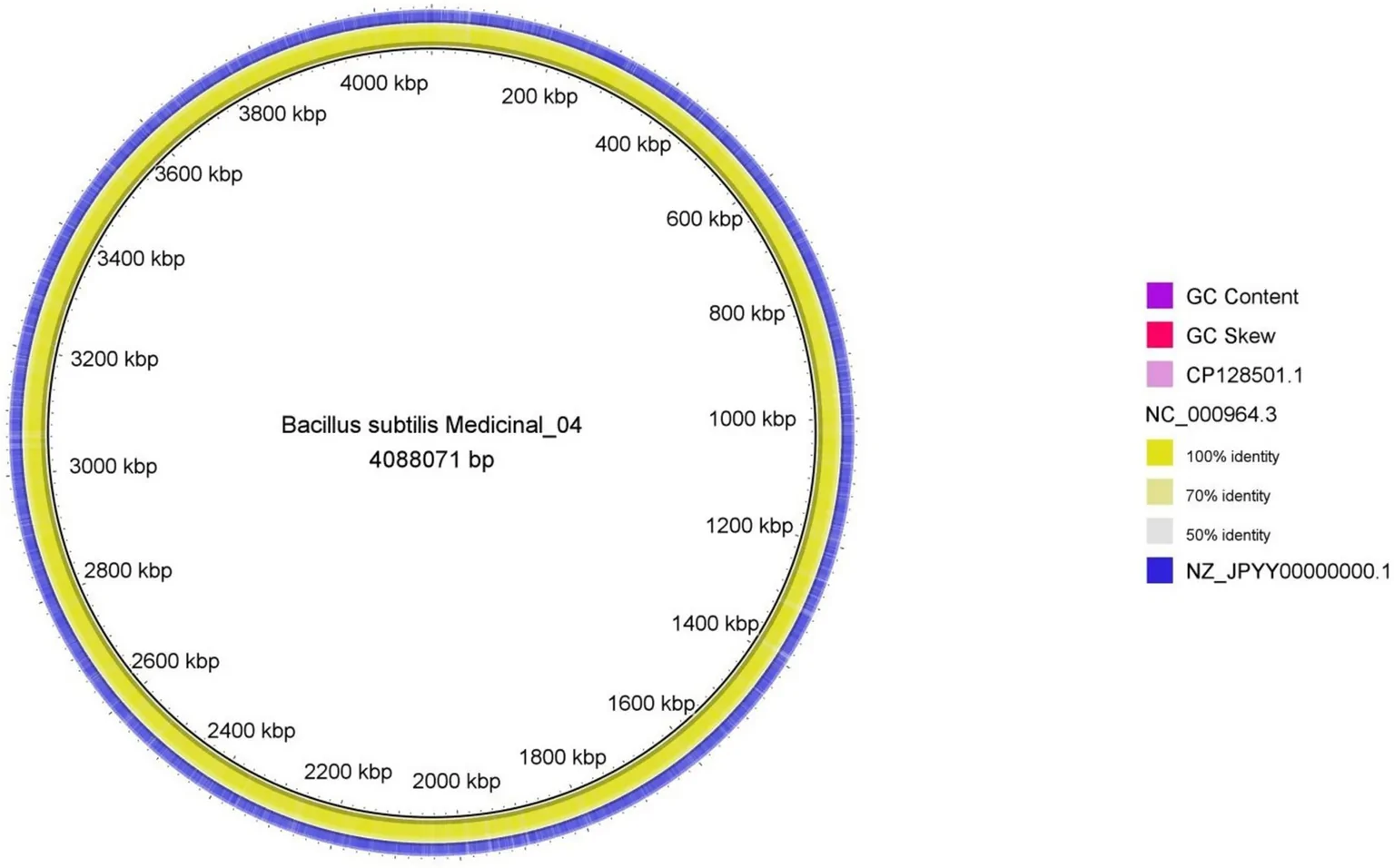
Blast Ring Image Generator (BRIG) map of *B. subtilis* subsp. *subtilis* str. 168, *B. velezensis* strain NKYL29 (accession no: NZ_JPYY00000000.1), and *B. amyloliquefaciens* strain SRCM123386 (accession no: CP128501.1) genomes. The query genome was aligned with three reference genomes using BRIG, which displays percent G + C, GC skew, and homology based on BLASTn (from inner to outer). Color saturation indicates the homology rate, and blanks (white regions) show the absence of similarity. *Bacillus subtilis* subsp. *subtilis* str. 168 is used as a reference strain.

To visually resolve the taxonomic positioning of the isolate within the *Bacillus subtilis* species complex, a blast-based comparative circular genome map was generated using the Blast Ring Image Generator (BRIG) (Figure 1). The complete chromosome of our isolated strain, *B. subtilis* Medicinal_04 (~4.08 Mb), served as the query sequence. It was aligned against the reference genomes of *B. subtilis* subsp. subtilis 168 (NC_000964.3), *B. velezensis* (NZ_JPYY00000000.1), and *B. amyloliquefaciens* (CP128501.1) to analyze structural similarities. The concentric alignment revealed striking differences in macro-synteny and sequence conservation across the three complex species. The alignment ring for *B. subtilis* subsp. subtilis str. 168 (NC_000964.3) displayed a solid, uninterrupted yellow profile representing ~100% sequence identity across the entire chromosome. Conversely, the outer blue concentric ring corresponding to *B. velezensis* (NZ_JPYY00000000.1) exhibited widespread gaps, severe thinning, and fragmentation, indicating sharp declines in sequence identity across multiple genomic loci. Furthermore, the concentric ring for *B. amyloliquefaciens* (CP128501.1) is largely absent due to falling below the identity visualization threshold. These structural differences visually confirm that the query genome (*B. subtilis* Medicinal 04) shares high genomic synteny and definitive species boundaries exclusively with *B. subtilis*.

The secondary metabolite biosynthetic gene clusters of the *B. subtilis* Medicinal 04 and reference *B. subtilis* subsp. subtilis str. 168 were compared and evaluated to explore the genetic basis underlying the functional antimicrobial traits conferred by *B. subtilis* JBWWSQ000000000. Genome mining via the antiSMASH 8.4.0 pipeline under relaxed parameters (Blin et al., 2025) identified a highly conserved core antimicrobial array between the query isolate *Bacillus subtilis* Medical_04 (JBWWSQ000000000) and the reference strain *B. subtilis* subsp. *subtilis* str. 168 (NC_000964.3), directly highlighting the strain’s potential as a powerful biocontrol agent and plant-growth-promoting rhizobacterium (PGPR) (Table 3). The high-confidence clusters for fengycin (Query Region 38.1), bacilysin (Query Region 20.1), and pulcherriminic acid (Query Region 18.1) were intact across both genomes. Fengycin serves as a powerful lipopeptide that targets and disrupts fungal cell membranes (Bender et al., 2026), while bacilysin drives broad-spectrum antibacterial activity (Islam et al., 2022), and pulcherriminic acid acts as an iron-chelating agent that starves out localized rhizosphere pathogens (Srinivasan et al., 2026). Additionally, the broad-spectrum antibacterial polyketide bacillaene was successfully captured in the query assembly, though fragmented across Regions 7.1, 118.1, and 168.1. The fitness-related sporulation killing factor involved in population survival and niche maintenance was also found conserved with medium confidence (Region 24.1).

**Table 3:** Gene clusters encoding secondary metabolites in *B. subtilis* Medicinal 04 using antiSMASH.

Biosynthetic target compound	Reference 168 region (confidence)	Query genome region (confidence)	Comparative interpretation and status
Fengycin (antifungal)	Region 5 (high)	Region 38.1 (high)	Intact and highly conserved across both strains.
Bacilysin (antibacterial)	Region 13 (High)	Region 20.1 (high)	Intact and highly conserved across both strains.
Pulcherriminic acid (iron chelation)	Region 11 (High)	Region 18.1 (high)	Intact and highly conserved across both strains.
Sporulation killing factor	Region 1 (high)	Region 24.1 (medium)	Conserved fitness/cannibalism machinery.
Bacillaene (antibacterial polyketide)	Region 4 (high)	Regions 7.1, 118.1, 168.1 (Low)	Single continuous cluster in Ref; fragmented across contigs in Query draft assembly.
Surfactin (biofilm/motility)	Region 2 (high)	Region 99.1 (low)	Query harbors a sequence-divergent wild-type variant distinct from domesticated 168.
Subtilosin A (bacteriocin)	Region 12 (high)	Region 203.1 (low)	Conserved bacteriocin variant; localized divergence in query assembly.
Plipastatin (antifungal)	___	Regions 101.1, 303.1 (low)	Distinctly annotated in Query genome; fragmented across contigs.
Sublancin 168 (bacteriocin)	Region 7 (high)	Absent	Lineage-specific cluster unique to domesticated reference strain 168.

Prominent evolutionary divergence was identified within the specialized accessory lipopeptide and bacteriocin tracks, revealing key wild-type advantages of the query isolate over the domesticated laboratory reference (Table 3). While the reference strain 168 carries a classic high-identity surfactin operon (Region 2) rendered non-functional by historical laboratory cultivation mutations, the query genome hosts a sequence-divergent surfactin variant (Region 99.1, low database similarity). This wild-type variation is essential for functional biofilm matrix production, motility, and competitive root colonization in open soil ecosystems. Furthermore, the query genome exhibits structural variation in its bacteriocin profile; the reference-specific cluster sublancin 168 (Region 7) is completely absent, whereas the powerful antifungal lipopeptide plipastatin (Regions 101.1 and 303.1) is uniquely present in the query genome. A structural variant of the antibacterial bacteriocin subtilosin A was also detected (Region 203.1). Collectively, the specialized composition of intact core lipopeptides, functional wild type surfactin, and auxiliary plipastatin equips *B. subtilis* JBWWSQ000000000 with the necessary genetic machinery for persistent environmental fitness, ecological dominance, and targeted phytopathogen antagonism. The complete secondary metabolite biosynthetic gene clusters from antiSMASH 8.4.0 are provided in Supplementary Figures S1, S2.

### 3.3 *In vitro* antagonistic activity of *Bacillus subtilis* Medicinal_04 against *Cercospora canescens*

**Figure 2:**
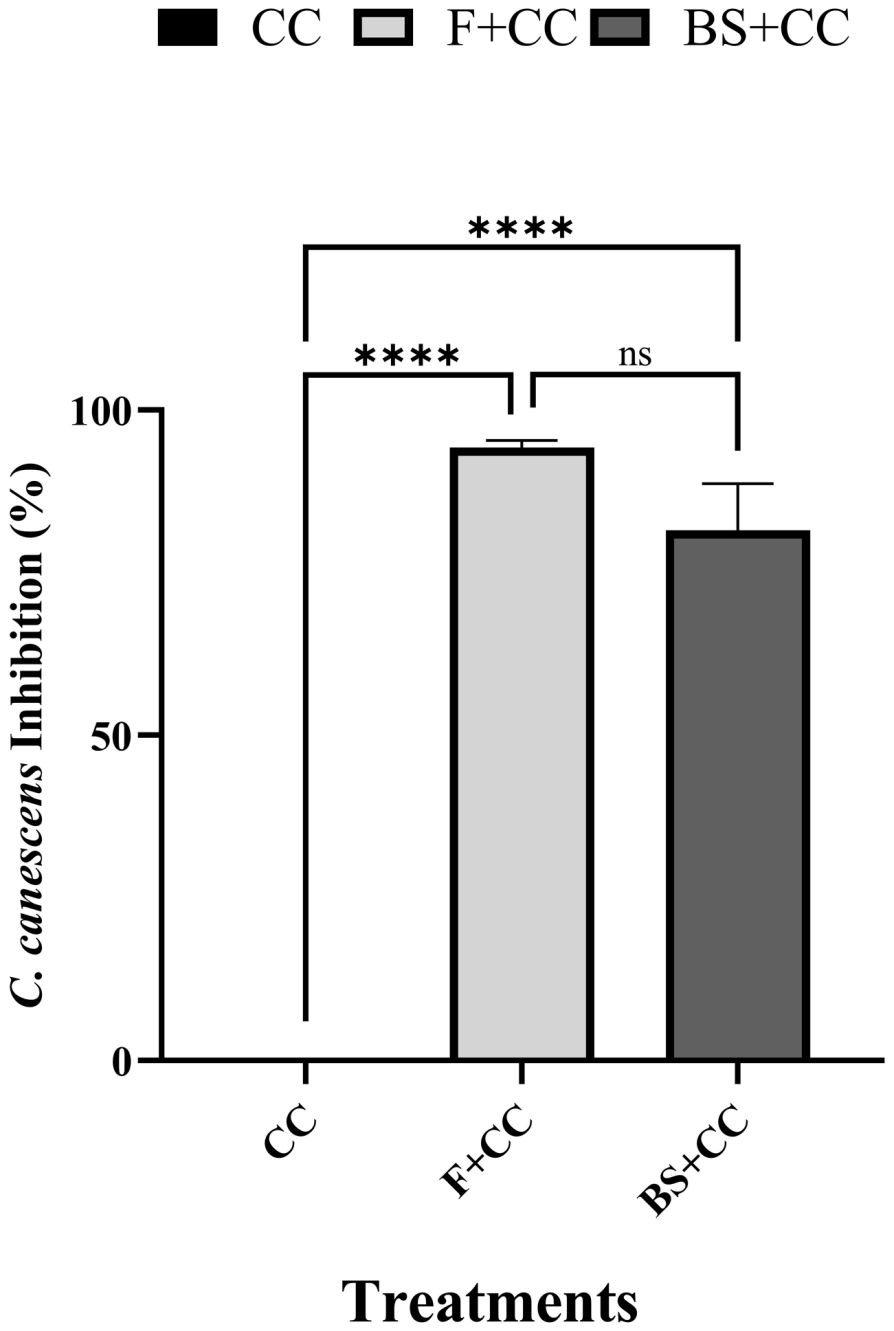
*In vitro* antifungal assay. *Cercospora canescens* (CC) growth inhibition (%) was measured at 7th day with or without treatments. CC = only *C. canescens*, F + CC = fungicide + *C. canescens*, BS + CC = *B. subtilis* + *C. canescens*. One-way ANOVA and Tukey multiple comparison test were used to test data statistically. Data are presented as means of three biological replicates and bars represent standard errors. The asterisks (*) indicate statistically significant differences, i.e., ****p < 0.0001, and ns = *p* > 0.05.

As established in the previous section that *B. subtilis* Medicinal_04 isolate, equipped with desired antimicrobial properties, has emerged as a potent biocontrol. These results are consistent with several previous studies in which *B. subtilis* strains were reported to inhibit fungal growth in cucumber and pepper (Cao et al., 2011; Qiao et al., 2023; Yang et al., 2024). Driven by these established mechanisms, the current study evaluated the direct antagonistic impact of *B. subtilis* Medicinal_04 against the destructive leaf spot pathogen *Cercospora canescens* using an *in vitro* disc-diffusion assay. The *C. canescens* growth was significantly reduced in the presence of *B. subtilis* and fungicide (Fungicide) compared to the mock-treated negative control (Figure 2). The quantitative analysis revealed that *B. subtilis* Medicinal_04 robustly restricted the radial growth of *C. canescens* by 81.5%, while the positive chemical control achieved a maximum inhibition rate of 94.0%. Although the synthetic fungicide demonstrated the highest absolute efficacy under laboratory conditions, *B. subtilis* Medicinal_04 displayed highly competitive, statistically significant antifungal potential, confirming its capability to effectively disrupt pathogen development *in vitro*.

### 3.4 In planta biocontrol efficacy against Cercospora leaf spot

**Figure 3:**
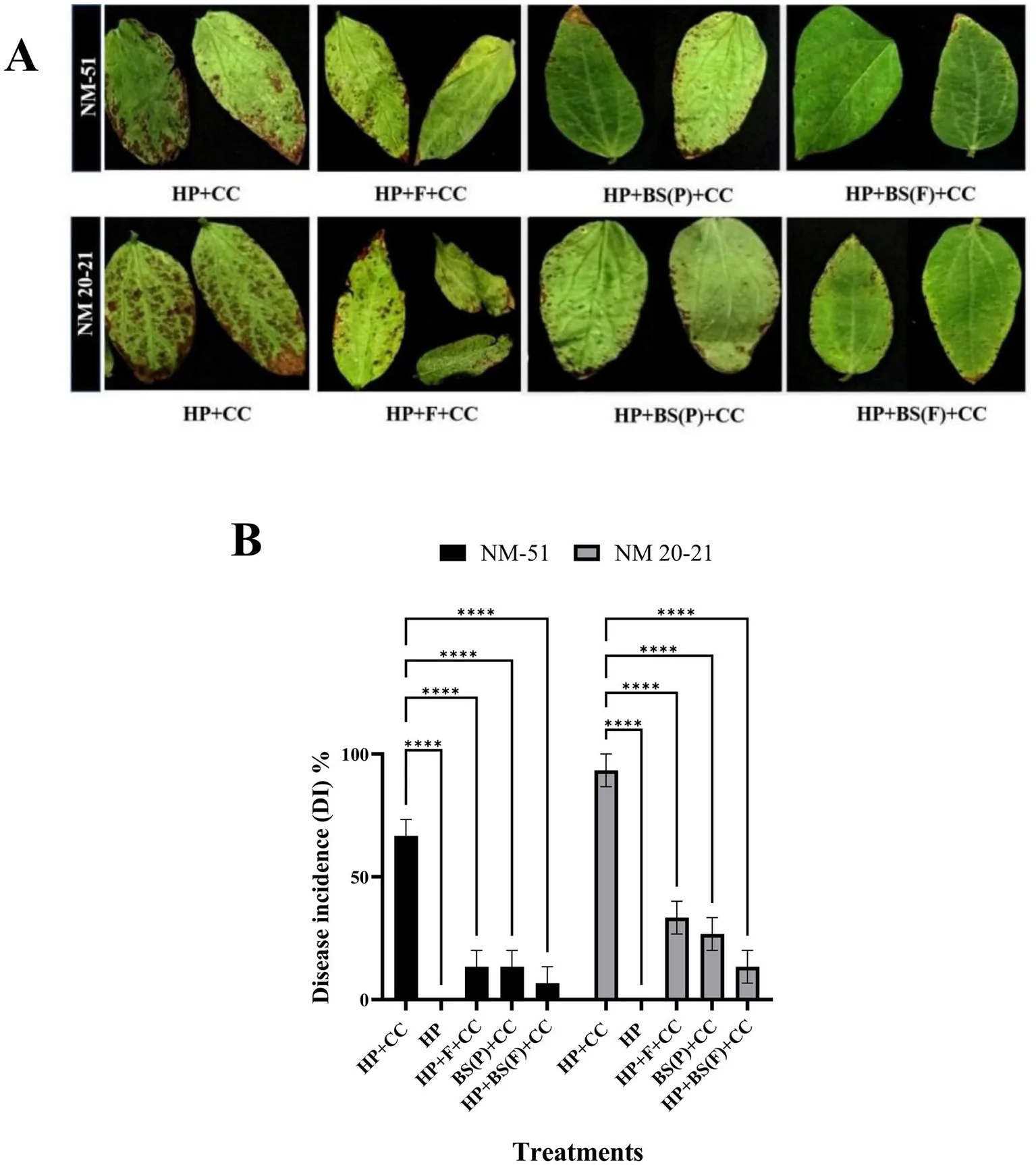
Effects of *B. subtilis* against CLS in mung bean varieties. **(A)** CLS disease prevalence across different treatment groups (30 DAS). **(B)** Disease incidence percentage (%) in response to different treatments. NM-20-21 and NM-51 mung bean varieties were used in this study. HP + CC (Positive Control) = Hydro-primed + foliar-application of *C. canescens*, HP (negative control) = Hydro-primed, HP + F + CC = Hydro-primed + fungicide + foliar-application of *C. canescens*, BS(P) + CC = *B. subtilis*-primed + foliar-application of *C. canescens*, HP + BS(F) + CC = Hydro-primed + *B. subtilis*-foliar-application + foliar-application of *C. canescens*. Two-way ANOVA and Tukey’s multiple comparison test were applied to test data statistically. All treatments are compared with HP + CC. Data are presented as means of three biological replicates and bars represent standard errors. The asterisks (*) indicate statistically significant differences, i.e., ****p < 0.0001.

Based on abovementioned *in vitro* bioassay results and fungal growth reduction, this led to hypothesizing whether the application of *B. subtilis* would successfully mitigate Cercospora leaf spot disease in mung bean grown in pots filled with soil. To evaluate the efficacy of *B. subtilis* against CLS NM-20-21 (CLS-susceptible) and NM-51 (CLS-resistant) mung bean varieties were subjected to either seed priming with *B. subtilis*, or foliar application of *B. subtilis*. Disease incidence (DI) was measured 30 DAS. Plants stressed with fungus alone (HP + CC) induced severe disease symptoms (Figure 3A), resulting in DI of 66% in the resistant variety NM-51 and a stark 93% in the susceptible variety NM-20-21 varieties, respectively (Figure 3B). Conversely, the fungicide control (HP + F + CC) significantly mitigated the infection, indicating disease reduction percentage (DRP) of 80 and 64% in NM-51 and NM-20-21, respectively (Figure 3). In contrast, biological antagonistic interventions such as seed priming with *B. subtilis* [BS(P) + CC] significantly suppressed infection reducing the DI by 71.4% in NM-20-21 and 80% in NM-51. The most profound suppression was achieved by foliar spray application [HP + BS(F) + CC], which reduced the DI by 85.7 and 90% across the same varieties NM-20-21 and NM-51, respectively. The representative photographs at 30 DAS (Figure 3A) visually demonstrate marked reductions in lesion development following *B. subtilis* treatments, particularly foliar application, consistent with the observed reductions in disease incidence. Collectively, these data demonstrate that while chemical fungicide application remains a potent standard for controlling CLS, biological treatments—particularly foliar application of *B. subtilis* Medicinal_04 provide an equally effective, eco-friendly alternative for establishing high-level disease reduction across both resistant and susceptible mung bean varieties.

### 3.5 Effect of *Bacillus subtilis* on plant morphological and physiological phenotypes under CLS stress

**Figure 4:**
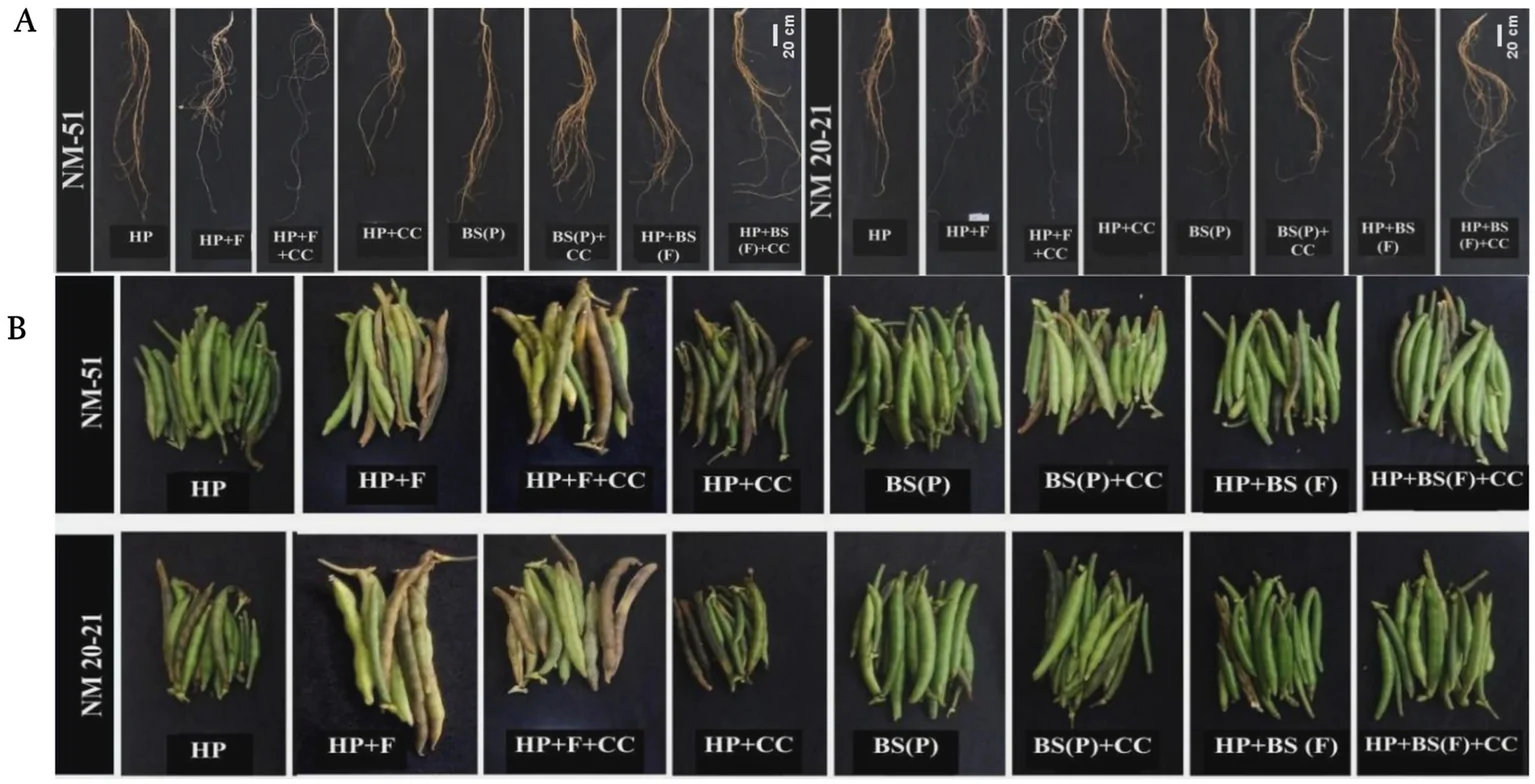
Root and pods harvested from treated plants. **(A)** Shows root length (pictures were captured using a reference scale to ensure accurate quantification of the growth parameters) while **(B)** shows number of pods among all treatments of NM-20-21 and NM-51 mung bean varieties. HP (negative control) = hydro-primed, HP + CC (positive control) = hydro-primed + foliar-application of *C. canescens*, HP + F + CC = hydro-primed + fungicide + foliar-application of *C. canescens*, BS(P) = *B. subtilis*-primed, BS(P) + CC = *B. subtilis*-primed + foliar-application of *C. canescens*, HP + BS(F) = hydro-primed + *B. subtilis*-foliar-application, HP + BS(F) + CC = hydro-primed + *B. subtilis*-foliar-application + foliar-application of *C. canescens*.

**Figure 5:**
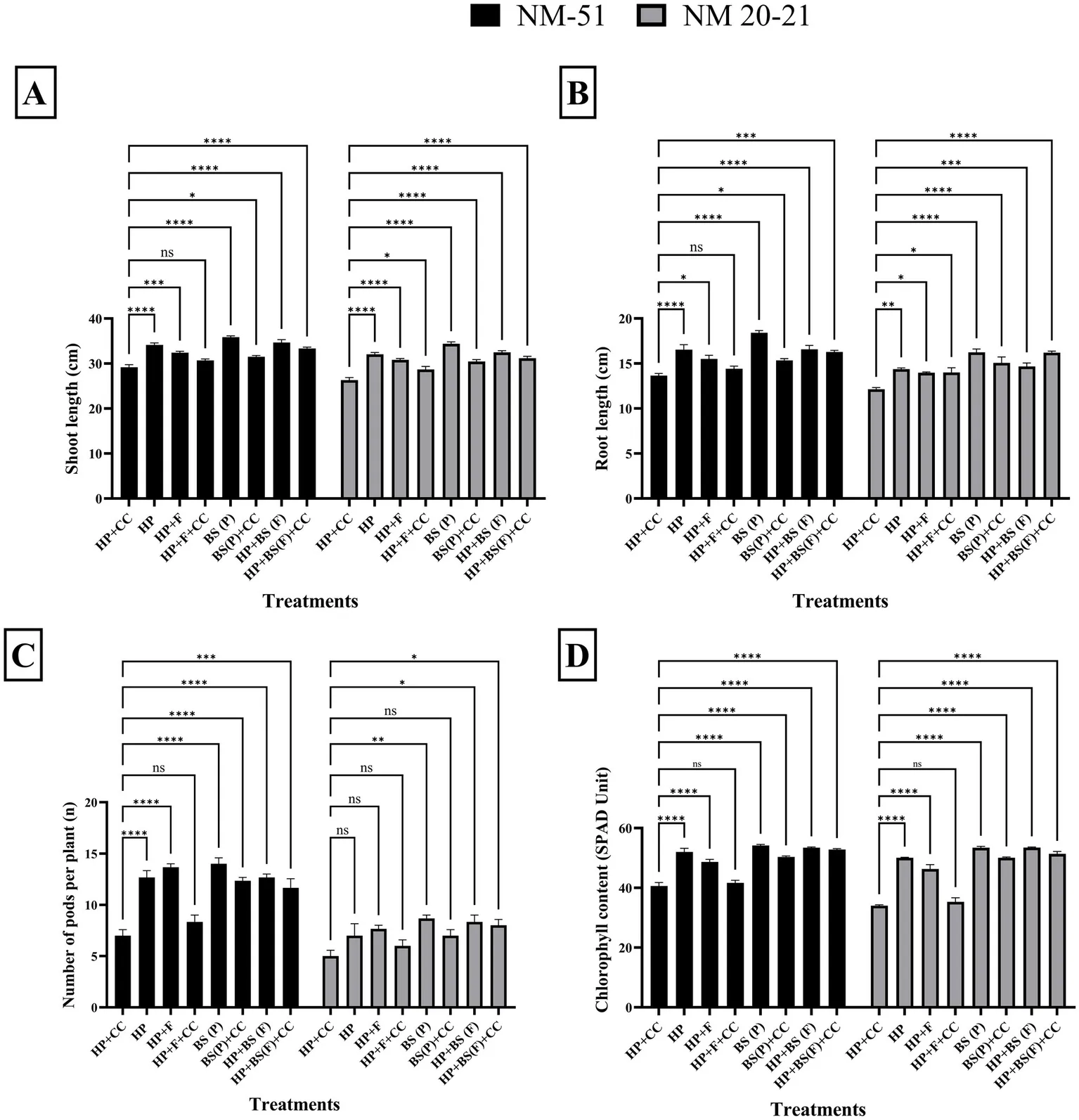
Effects of *B. subtilis* on shoot length **(A)**, root length **(B)**, number of pods **(C)**, and chlorophyll content **(D)** among all treatments. *B. subtilis* was evaluated in NM-51 and NM-20-21 mung bean varieties under CLS stress using seed priming and foliar-application. HP + CC (Positive Control) = Hydro-primed + foliar-application of *C. canescens*, HP (negative Control) = Hydro-primed, HP + F = Hydro-primed + foliar-application of fungicide, HP + F + CC = Hydro-primed + fungicide + foliar-application of *C. canescens*, BS(P) = *B. subtilis*-primed, HP + BS(F) = Hydro-primed + *B. subtilis*-foliar-application, BS(P) + CC = *B. subtilis*-primed + foliar-application of *C. canescens*, HP + BS(F) + CC = Hydro-primed + *B. subtilis*-foliar-application + foliar-application of *C. canescens*. Statistical analysis utilized two-way ANOVA and Tukey’s test. All treatments are compared with HP + CC. Data are presented as means of three biological replicates and bars represent standard errors. The asterisks (*) indicates statistically significant differences and levels, i.e., *p < 0.05, **p < 0.01, ***p < 0.001, ****p < 0.0001, and ns, *p* > 0.05.

Smaller pods, short roots and shoots, decreased number of pods and chlorophyll contents are reported to be common consequences of CLS disease (Rafiq et al., 2022), thus these agronomic and physiological parameters were quantified across both mung bean varieties. The study reveals that plants inoculated with pathogen alone (HP + CC) experienced 14.5 and 17.8% reductions in shoot length, 17.4 and 15.5% declines in root length, and plummets of 21.9 and 32.1% in total chlorophyll content in NM-51 and NM–20-21 varieties compared to controls (HP) (Figures 4, 5). The chemical fungicide treatment (HP + F + CC) showed an increase in shoot length by 5 and 7%, root length by 5 and 15%, number of pods by 19 and 20%, and total chlorophyll content by 2 and 3% in NM-51 and NM–20-21 varieties, respectively, compared to pathogen inoculated (HP + CC) however they showed reduced shoot and root lengths when compared to control (HP). Furthermore, fungicide spray on healthy plants (HP + F) induced minor phytotoxic depression over HP plants, showing 5 and 4% reduction in shoot length, 6 and 3% in root length, and 6 and 8% in chlorophyll content in NM 51 and NM-20-21 across the respective varieties compared to control (HP).

In the absence of the pathogen, seed priming with the biological antagonist (BS(P)) acted as a growth promoter compared to HP, increasing shoot length by 5.0 and 7.0%, root length by 12.0 and 13.0%, pod count by 10.0 and 24.0%, and chlorophyll accumulation by 4.0 and 7.0%. Conversely, healthy plants treated with the biological foliar spray alone (HP + BS(F)) exhibited statistically non-significant variations compared to HP. Exogenous foliar application of the antagonist (HP + BS(F) + CC) robustly mitigated CLS stress compared to the HP + CC baseline, triggering improvements in shoot length (14.2 and 18.5%), root length (19.2 and 33.5%), and chlorophyll content (30.6 and 51.1%) in NM-51 and NM-20-21, respectively. Similarly, seed priming under pathogen challenge (BS(P) + CC) enhanced shoot length by 8.0 and 15.7%, root length by 12.3 and 24.1%, and chlorophyll levels by 24.0 and 47.0% over HP + CC (Figures 4, 5).

Collectively, these results demonstrate that application of *B. subtilis* Medicinal_04 has improved morphological and physiological traits of mung bean under CLS stress. Crucially, the biological treatments outperformed standard chemical fungicide applications by avoiding minor chemical-induced growth depressions and promoting superior overall plant health. The data of morphological and physiological parameters of mung bean varieties (NM-51 and NM-20-21) in mean and standard error (SE) across different treatments are mentioned in Supplementary Table S3.

### 3.6 Effect of *Bacillus subtilis* on biochemical parameters

**Figure 6:**
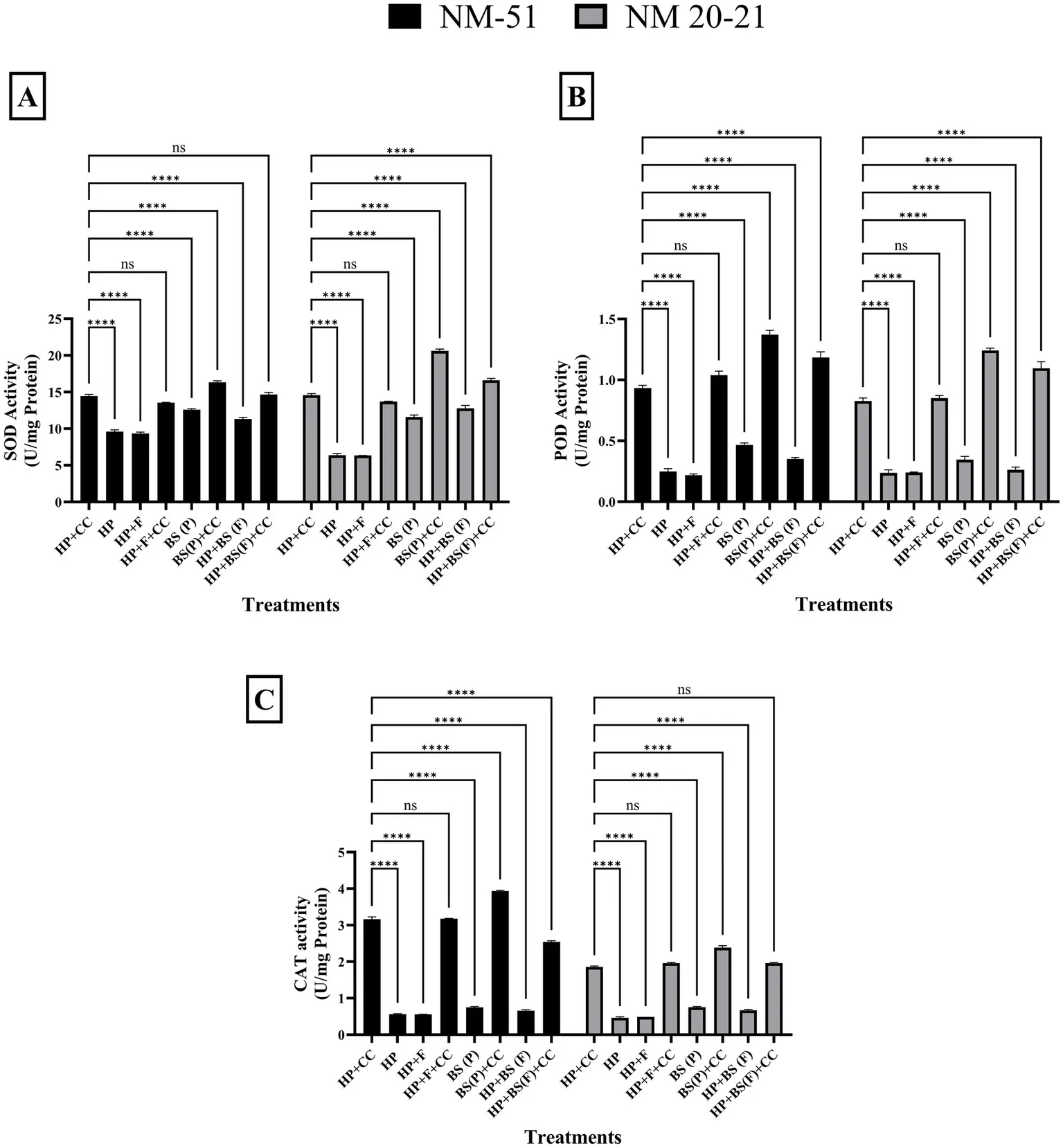
Effect of *B. subtilis* on SOD **(A)**, CAT **(B)**, POD **(C)** among all treatments: *B. subtilis* was evaluated in NM-51 and NM-20-21 mung bean varieties under CLS stress using seed priming and foliar application. HP + CC (Positive Control) = Hydro-primed + foliar-application of *C. canescens*, HP (Negative Control) = Hydro-primed, HP + F = Hydro-primed + foliar-application of fungicide, HP + F + CC = Hydro-primed + fungicide + foliar-application of *C. canescens*, BS(P) = *B. subtilis*-primed, HP + BS(F) = Hydro-primed + *B. subtilis*-foliar-application, BS(P) + CC = *B. subtilis*-primed + foliar-application of *C. canescens*, HP + BS(F) + CC = Hydro-primed + *B. subtilis*-foliar-application + foliar-application of *C. canescens*. Two-way ANOVA and Tukey’s multiple comparison test were applied to test data statistically. All treatments are compared with HP + CC. Data are presented as means of three biological replicates and bars represent standard errors. The asterisks (*) indicate statistically significant differences and levels, i.e., ****p < 0.0001 and ns = *p* > 0.05.

Cercospora leaf spot disease typically triggers an accumulation of reactive oxygen species (ROS) in host cells, promoting the activation of its antioxidant enzymes defense system to scavenge these oxidants (Choudhary and Chand, 2022). Thus, antioxidants such as superoxide dismutase (SOD), peroxidase (POD), and catalase (CAT) activities were quantified across both varieties to evaluate how our treatments modulate plant defense network (Figure 6; Supplementary Table S4). As shown in Figure 6, challenging the host with the pathogen alone (HP + CC) showed a significant 1.5-fold and 2.28-fold increase in SOD, 4.5-fold and 4-fold in POD and 5.3-fold and 3.8-fold CAT in NM 51 and NM − 20-21 varieties respectively, as compared to their respective control (HP). Compared to control (HP), the healthy plants applied with fungicide (HP + F plants) showed statistically indifferent results in CAT, POD and SOD activities in both varieties. Similarly, fungicide spray under disease pressure (HP + F + CC) displayed non-significant results in CAT, POD and SOD in both varieties compared to HP + CC cohort.

In the absence of the pathogen, seed priming with the biological antagonist (BS(P)) significantly boosted antioxidant production, elevating SOD (1.31-fold and 1.81-fold), POD (2.5-fold and 1.5-fold) and CAT (1.17-fold and 1.6-fold) fold change compared to HP plants. Similarly, healthy plants with biological foliar spray alone HP + BS(F) demonstrated a substantial increase in SOD (1.18-fold and 2-fold), POD (2-fold and 1.5-fold) and CAT (1.17-fold and 1.4-fold). When subjected to pathogen pressure, the biological treatments further accelerated the host’s antioxidant response over the HP + CC baseline. Combining seed priming with the pathogen challenge (BS(P) + CC) significantly enhanced SOD (1.13-fold and 1.41-fold), POD (1.56-fold and 1.5-fold) and CAT (1.22-fold and 1.26-fold) activities across the respective varieties. Under the same disease challenge, foliar application (HP + BS(F) + CC) also showed a significant increase in SOD (1.02-fold and 1.14-fold), and POD (1.33-fold and 1.38-fold) compared to HP + CC in NM-51 and NM–20-21 varieties, respectively, while its impact on CAT levels remained statistically non-significant for NM–20-21 variety. Collectively, these findings demonstrate that *B. subtilis* Medicinal_04 application primes and enhances the host biochemical machinery under CLS stress, reinforcing the enzymatic scavenging network to successfully mitigate oxidative cellular damage.

### 3.7 Effect of *Bacillus subtilis* on *PR-1* gene expression

**Figure 7:**
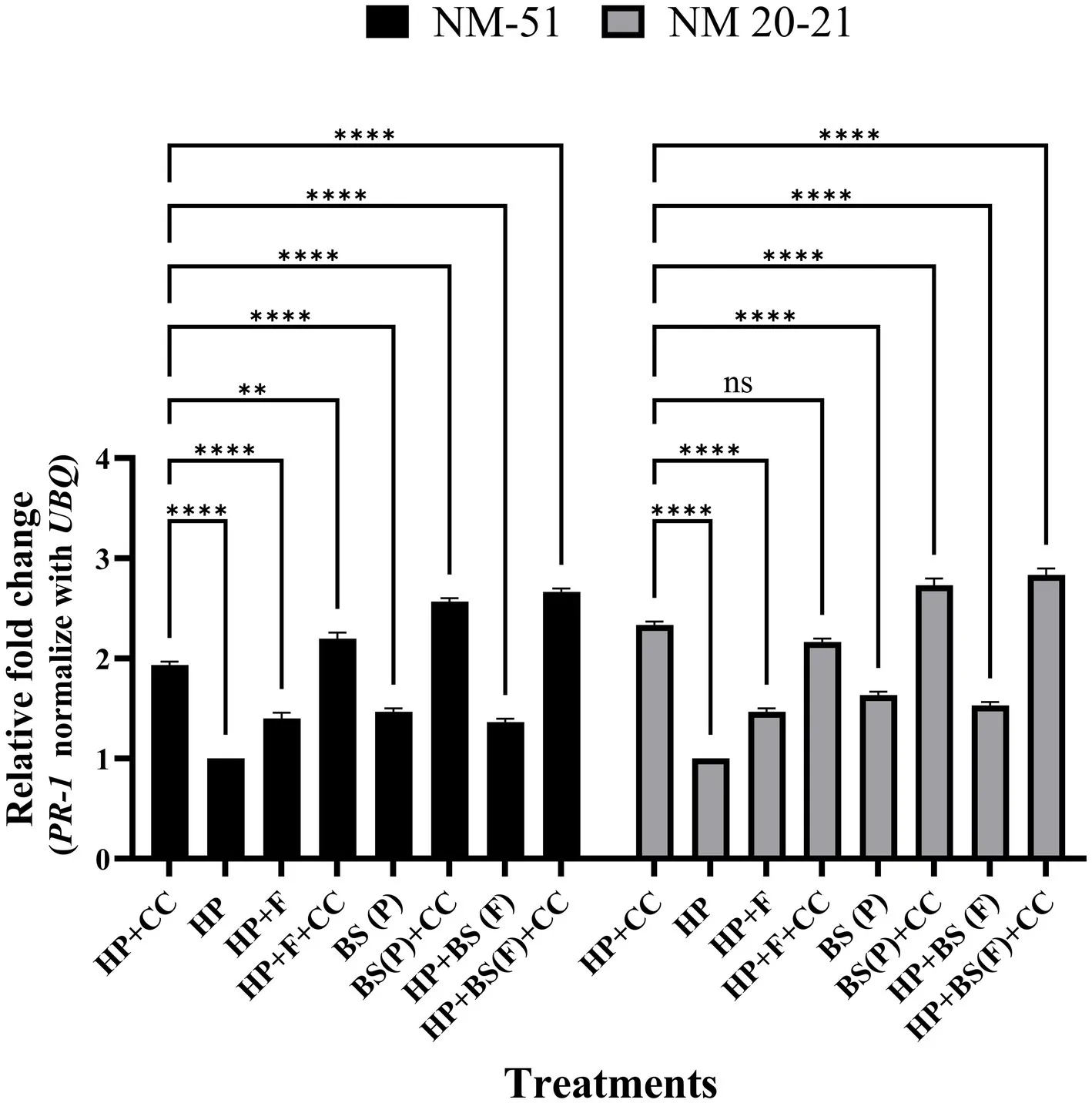
*PR-I e*xpression analysis among all treatments: this figure shows regulation status of pathogenesis-related gene in mung bean varieties NM-20-21 and NM-51, studied for the impact of *B. subtilis* under CLS stress. HP + CC (Positive Control) = Hydro-primed + foliar-application of *C. canescens*, HP (Negative Control) = Hydro-primed, HP + F = Hydro-primed +foliar-application of fungicide, HP + F + CC = Hydro-primed + fungicide + foliar-application of *C. canescens*, BS(P) = B. subtilis-primed, HP + BS(F) = Hydro-primed + B. subtilis-foliar-application, BS(P) + CC = B. subtilis-primed + foliar-application of *C. canescens*, HP + BS(F) + CC = Hydro-primed + B. subtilis-foliar-application + foliar-application of *C. canescens*. Two-way ANOVA and Tukey’s test evaluated data. All treatments were compared to HP + CC. Data are presented as means of three biological replicates and bars represent standard errors. The asterisks (*) indicate statistically significant differences and levels, i.e., **p < 0.01, ****p < 0.0001, and ns = *p* > 0.05.

*Pathogenesis-related (PR-1)* genes are key molecular markers for host defense and Systemic Acquired Resistance (SAR) activation during fungal invasion (Yadav et al., 2024). To determine if the antagonist transcriptionally primes the host, *PR-1* expression dynamics were monitored via real-time qRT-PCR across both varieties (Figure 7; Supplementary Table S4). As shown in Figure 7, stressing the host with the pathogen alone (HP + CC cohort) triggered a sharp transcriptomic stress response, significantly upregulating *PR-1* expression by 1.93-fold and 2.33-fold in NM 51 and NM-20-21 varieties, respectively, compared to the HP. The standalone treatments without the pathogen also revealed enhanced gene induction relative to HP: seed priming BS(P) increased *PR-1* levels by up to 1.63-fold, followed by the biological foliar spray (HP + BS(F)) at 1.53-fold, and the chemical fungicide baseline (HP + F) at 1.47-fold.

Under active disease pressure, co-application of *B. subtilis* Medicinal_04 further amplified defense gene transcripts. When compared against the HP + CC baseline, both the combined foliar application (HP + BS(F) + CC) and the seed-priming (BS(P) + CC) cohorts induced further upregulated *PR-1* expression in both varieties. However, the chemical fungicide application (HP + F + CC) treatment showed a mixed response: it upregulated *PR-1* expression by 1.14-fold in NM-51 but showed down-regulation in the NM-20-21 variety (Figure 7). Therefore, it can conclude that application of *B. subtilis* acts as a potent biological elicitor, with enhanced *PR-1* expression under active CLS stress to fortify host systemic defenses and suppress disease progression.

## 4 Discussion

Cercospora Leaf Spot (CLS), caused by *Cercospora canescens*, poses a severe threat to mung bean production in Pakistan, typically driving severe yield losses up to 95% under favorable epidemic conditions (Abbas et al., 2020). While intensive synthetic chemical fungicide applications remain a common standard for managing CLS, they compromise food safety, cause environmental pollution, and trigger phytotoxic stress that limits host development (Abbas et al., 2020; Said, 2023). Plant growth-promoting rhizobacteria (PGPR) have attracted increasing attention as environmentally sustainable alternatives because they can simultaneously suppress plant pathogens and enhance crop growth like *Amycolatopsis* sp. SND-1 and *Bradyrhizobium* sp. DOA9. However, many candidate biocontrol agents fail to transition effectively from *in vitro* screens to complex field environments due to environmental sensitivity or narrow host ranges (Basavarajappa et al., 2023; Songsaeng et al., 2024). Consequently, recruiting spore-forming, robust biocontrol agents capable of enduring environmental fluctuations while maintaining high antagonistic efficacy is essential (Wang et al., 2025). The current study addresses this gap by demonstrating that the multi-functional rhizospheric isolate *Bacillus subtilis* Medicinal_04 effectively suppresses CLS disease while concurrently acting as a potent bio-stimulant under active pathogen pressure.

The biocontrol capability of *B. subtilis* Medicinal_04 was initially evidenced by an 81.5% inhibition of *C. canescens* mycelial growth in radial diffusion assays. This direct antagonism correlates with the strain’s pronounced chitinolytic activity (++), which mediates the enzymatic degradation of fungal cell wall chitin (Saini et al., 2024). *In silico* antiSMASH genome mining provided molecular foundation for these phenotypic observations, revealing an extensive secondary metabolite biosynthetic gene cluster (BGC) repertoire. This includes BGCs encoding lipopeptides (surfactin, iturin, fengycin, plipastatin), polyketides (bacillaene), dipeptides (bacilysin), antimicrobial peptides (subtilosin A, sporulation killing factor), and bacillibactin (catecholate siderophore). Comparative genomic analysis demonstrated that while the query genome lacks the reference-specific sublancin 168 cluster (Region 7), it uniquely harbors the potent antifungal plipastatin locus (Regions 101.1 and 303.1) alongside fengycin BGCs. The co-occurrence of fengycin and plipastatin pathways provides a synergistic mechanism for permeabilizing fungal lipid membranes, directly supporting the observed biofungicidal efficacy against *C. canescens* (Alam et al., 2025; Bender et al., 2026). These genomic features provide molecular support for the observed biocontrol activity of *B. subtilis* Medicinal_04 against *C. canescens*.

In addition to direct antagonism, genome mining and phenotypic profiling confirmed that *B. subtilis* Medicinal_04 possesses key traits for plant growth promotion and nutritional competition. The strain exhibited strong catalase activity (+), robust ammonia production (+), and indole-3-acetic acid (IAA) synthesis (+). Genomic mining further identified a high-confidence tRNA-dependent cyclodipeptide synthase (CDPS) cluster (Region 18.1) responsible for pulcherriminic acid biosynthesis, alongside the bacillibactin siderophore locus. These phenotypic and genomic features collectively indicate the potential of *B. subtilis* Medicinal_04 to enhance plant growth, improve iron acquisition, and compete effectively with phytopathogens (Srinivasan et al., 2026; Yang et al., 2025). Although inorganic phosphate solubilization was absent *in vitro*, these combined phytohormone and iron-acquisition pathways supplied sufficient biostimulatory support to enhance host fitness under pathogen challenge.

Evaluating disease dynamics across mung bean cultivars with varying resistance profiles validated the bio-control scope of *B. subtilis* Medicinal_04. The commercial cultivars NM-51 (generally classified as resistant or tolerant) and NM-20-21 (susceptible) (Ilyas et al., 2023; Nawaz et al., 2023) were evaluated under controlled, high-humidity artificial inoculation conditions. Previous field evaluations by Ilyas et al. (2023) reported lower disease severity in the resistant cultivar NM-51 (19.23%) than in the susceptible cultivar NM-20-21 (35.32%). Under the controlled inoculation conditions used in the present study, NM-51 likewise exhibited lower disease incidence (66%) than NM-20-21 (93%), indicating that the resistant genotype maintained greater tolerance even under high pathogen pressure. Although disease severity and disease incidence are different epidemiological parameters and cannot be directly compared, both studies consistently identified NM-51 as resistant and NM-20-21 as susceptible to *C. canescens*. As in the present study, plants were subjected to artificial inoculation under controlled conditions using a pathogen inoculum and disease-conducive environmental conditions (such as humidity) to ensure successful disease establishment. Thus, the 66% disease incidence observed in NM-51 under the HP + CC treatment reflects the incidence of the artificial disease challenge rather than a loss of genetic resistant and classification. Crucially, application of *B. subtilis* Medicinal_04 significantly mitigated CLS symptoms in both genetic backgrounds.

Comparative delivery regimes demonstrated that while seed priming (BS(P)) established strong early root development, exogenous foliar application (HP + BS(F) + CC) yielded stronger disease suppression, reducing CLS incidence by 90.0% in NM-51 and 85.7% in NM-20-21. Since *C. canescens* infects primarily through foliar tissues, direct application of the bacterial antagonist to the phyllosphere enables immediate interception of germinating fungal spores through localized secretion of lipopeptides and cell-wall degrading chitinases. However, there remains a notable lack of comparative literature regarding the relative effectiveness of seed priming and foliar application. This aligns with findings by Awan and Shoaib (2019), who reported a 67–83% reduction in *Alternaria solani* severity following foliar application of *B. subtilis*.

Beyond disease suppression, *B. subtilis* Medicinal_04 rescued host vegetative and reproductive growth under stress while avoiding the phytotoxic side effects of chemical fungicides. While synthetic fungicide application (HP + F) on uninoculated plants caused minor growth depression—reducing shoot length by 5.0% and chlorophyll content by 8.0% relative to control (HP)—biological treatments optimized growth metrics. In healthy plants, seed priming (BS(P)) increased root length by 13.0% and pod count by 24.0%. Under active stress, the foliar regime (HP + BS(F) + CC) achieved striking recovery, boosting shoot lengths by up to 18.5% and chlorophyll content by 51.1% over HP + CC. This increased shoot length and chlorophyll recovery is likely driven by enhanced iron bioavailability driven by pulcherriminic acid and bacillibactin production, alongside IAA-mediated physiological regulation (Jassim et al., 2025). Furthermore, the elevated chlorophyll levels indicate improved iron assimilation, a trait directly supported by the genomic identification of a high-confidence CDPS biosynthetic cluster (Region 18.1) tied to the pulcherriminic acid pathway. As a potent iron-chelating agent, pulcherriminic acid serves a dual role: satisfying host iron nutrition via biostimulation while concurrently starving localized pathogens of essential iron in the rhizosphere (Freitas et al., 2015; Jassim et al., 2025). Consequently, these outcomes strongly suggest that *B. subtilis* Medicinal_04 promotes plant development through the orchestration of the aforementioned phytohormone pathways and iron-acquisition mechanisms, though the exact signaling cascades warrant further experimental validation.

At the physiological and molecular levels, *B. subtilis* Medicinal_04 primed host endogenous immunity to neutralize pathogen-induced oxidative stress. *C. canescens* infection typically triggers excessive reactive oxygen species (ROS) accumulation, leading to membrane lipid peroxidation and cell collapse (Choudhary and Chand, 2022). Plants treated with *B. subtilis* exhibited significantly elevated antioxidant enzyme activities, increasing superoxide dismutase (SOD) by up to 1.41-fold and peroxidase (POD) by up to 1.56-fold compared to untreated infected controls. This enzymatic surge was complemented by the strain’s intrinsic catalase activity (+), which aids in scavenging extracellular ROS at the host–microbe interface (Hasanuzzaman et al., 2022). Furthermore, qRT-PCR analysis revealed strong transcriptional upregulation of the pathogenesis-related gene *PR-1*—a key marker for systemic acquired resistance (SAR) (Yadav et al., 2024). While chemical fungicide treatment (HP + F + CC) resulted in inconsistent defense gene expression, biological treatment induced consistent, multi-fold *PR-1* hyper-induction across both resistant and susceptible cultivars. This robust upregulation mirrors the systemic hormonal cascades reported by Rumyantsev et al. (2023), confirming that *B. subtilis* Medicinal_04 transcriptionally elicits host immune networks to halt pathogen progression.

**Figure 8:**
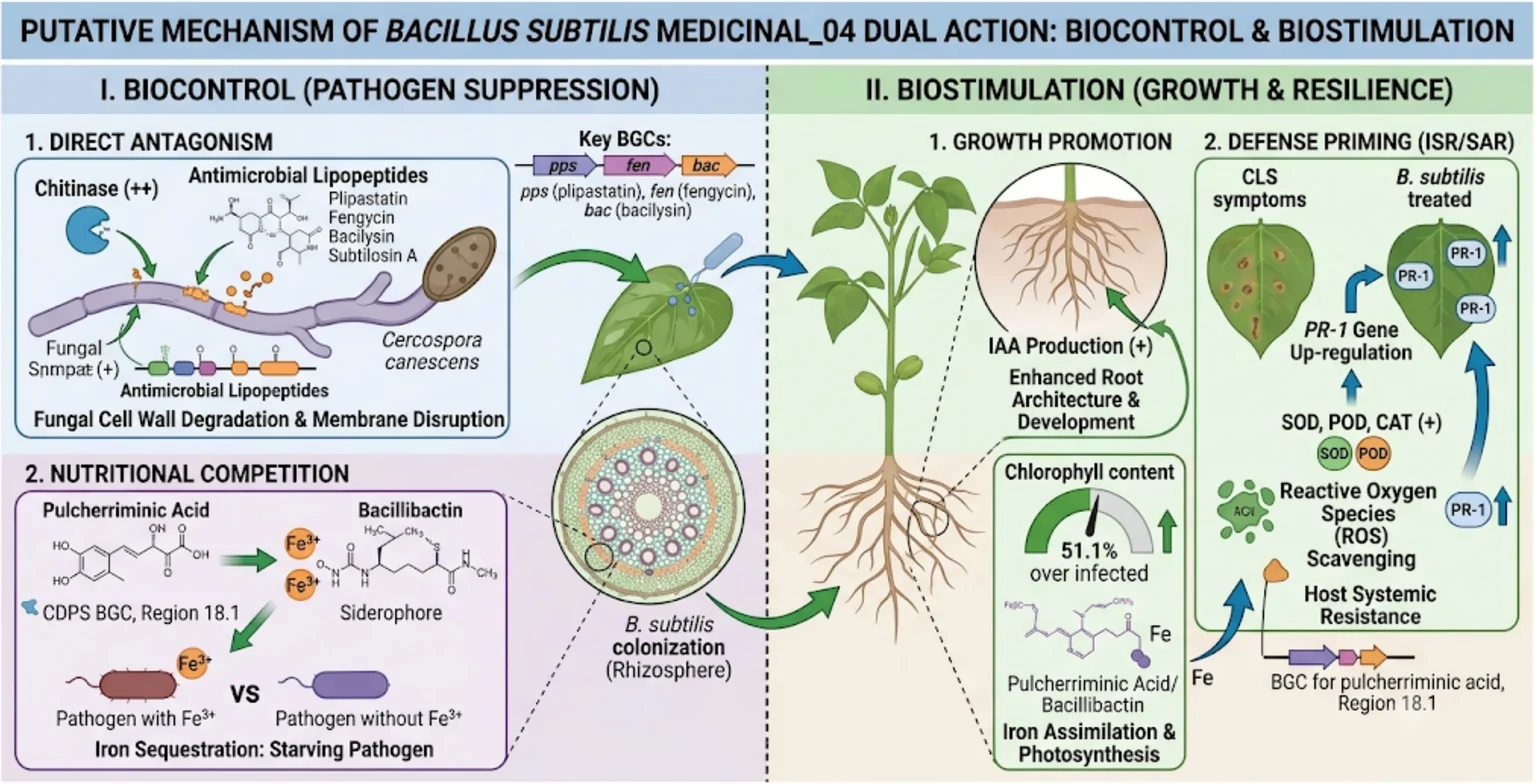
Putative dual mechanism model for *B. subtilis* Medicinal_04. This diagram integrates genomic data, biochemical assays, and physiological outcomes to illustrate how the multi-functional isolate manages Cercospora leaf spot (CLS) in mung bean while simultaneously promoting plant growth and resilience through direct antagonism, nutritional competition, biostimulation, and host defense priming pathways.

Integrating our genomic, biochemical, and physiological findings, we propose a comprehensive model illuminating how *B. subtilis* Medicinal_04 achieves simultaneous biocontrol and biostimulation in mung bean challenged by CLS (Figure 8). The mechanism operates through a powerful intersection of direct pathogen suppression and indirect host resilience pathways. Biocontrol is driven by direct antagonism—where secreted lipopeptides (e.g., fengycin, plipastatin), bacilysin, and chitinase enzymatically degrade *C. canescens* cells—and by nutritional competition, as the siderophore bacillibactin and pulcherriminic acid effectively sequester iron to starve the pathogen (Srinivasan et al., 2026). Concurrently, biostimulation is achieved through bacterial IAA production, which remodels root architecture for enhanced water and nutrient uptake, and improved iron assimilation (Yang et al., 2025), supported by the same iron-chelating BGCs, which boosted chlorophyll content by 51.1%. Crucially, the biological application systemically primes the host’s innate defense networks (ISR/SAR), marked by the robust upregulation of antioxidant enzymes (SOD, POD, CAT) to neutralize pathogen-induced ROS and the aggressive hyper-induction of *PR-1* transcripts, reinforcing host immunity across both resistant and susceptible cultivars to effectively halt pathogen progression.

In summary, the multi-faceted efficacy of *B. subtilis* Medicinal_04 operates through a combination of direct antibiosis and host-mediated defense priming. Direct fungal suppression is driven by chitinolytic degradation and membrane-disrupting lipopeptides (fengycin, plipastatin, bacilysin), while nutritional competition is governed by siderophore production (bacillibactin). Simultaneously, bacterial signaling elicits host defense networks via enhanced ROS-scavenging enzymes (SOD, POD, CAT) and upregulated *PR-1* transcript expression, characteristic of induced systemic resistance (ISR) and SAR pathways. Coupled with IAA-driven biostimulation, *B. subtilis* Medicinal_04 represents a robust, highly effective eco-friendly alternative to synthetic fungicides for managing Cercospora Leaf Spot in mung bean production.

### 4.1 Limitations

This study demonstrates the dual function of *B. subtilis* Medicinal_04 in promoting plant growth and suppressing Cercospora leaf spot under controlled lath house conditions while comparing the effectiveness of seed priming and foliar application. Using three biological replicates represents standard practice for space-constrained *in planta* screenings. However, a relatively small sample size of only three independent biological replicates (pots) per treatment and controlled conditions may have limited the generalizability of the findings. In addition, disease assessment was based on disease incidence, a binary measure that does not capture differences in symptom severity among infected plants. The study also did not evaluate different bacterial or spore concentrations, the long-term persistence of *B. subtilis* Medicinal_04 in soil, or its potential effects on native soil microbiota. Furthermore, although enhanced antioxidant enzyme activities and *PR-1* expression suggested activation of host defense responses, the underlying ISR/SAR pathways, phytohormone regulation, and *in planta* production of specialized metabolites were not directly investigated. Therefore, field validation under diverse environmental conditions is required before large-scale application.

### 4.2 Future directions

To build upon these foundational findings and facilitate the seamless transition of *B. subtilis* Medicinal_04 into real-world agricultural systems, several crucial research avenues must be explored. Future investigations should focus on extensive dose–response trials across a wide concentration gradient to optimize the precise spore load required for maximum disease control and economic feasibility. Additionally, open-field trials across diverse agro-ecological zones should be conducted to validate the long-term stability and persistence of the foliar and seed-priming regimes against wild pathogen pressures. Finally, to unravel the deeper molecular mechanics, *in planta* lipopeptide and hormone production should be quantified during active infection to understand antifungal peptides accumulation and signaling dynamics.

## 5 Conclusion

In conclusion, the integration of phylogenomic, biochemical, and *in planta* datasets demonstrates that *B. subtilis* Medicinal_04 is a highly effective alternative to synthetic fungicides. By combining direct chitinolytic antagonism and secondary metabolite production with host phytohormone modulation, antioxidant enzyme amplification, and *PR-1* transcriptional priming, this strain provides a comprehensive defense framework against *C. canescens*. The highest disease inhibition was recorded for the foliar application of *B. subtilis* Medicinal_04 against Cercospora leaf spot (CLS) disease, yielding 90.0 and 85.7% reduction in the NM-51 and NM-20-21 varieties, respectively. Thus, compared to seed priming, the foliar application of *B. subtilis* Medicinal_04 emerged as the most effective treatment for managing CLS disease in mung bean. Given its dual capacity for high-level disease suppression and direct plant growth promotion without phytotoxic trade-offs, *B. subtilis* Medicinal_04 represents a valuable candidate for large-scale application within climate-smart and sustainable agricultural systems.

## Data Availability

The datasets presented in this study can be found in online repositories. The names of the repository/repositories and accession number(s) can be found in the article/Supplementary material.

## Footnotes

1
https://microbesng.com/

2
https://getgenome.net/

3
https://jspecies.ribohost.com/jspeciesws/#analyse

4
https://www.ncbi.nlm.nih.gov/gene/106769349

5
https://perlprimer.sourceforge.net/download.html

6
https://www.graphpad.com/
