# Supplementary material for: Bacillus subtilis isolated from medicinal plants rhizosphere effectively controls Cercospora leaf spot and improves plant growth in mung bean (Vigna radiata)
Source: Front Microbiol. 2026 Aug 19;17:1850863. doi: 10.3389/fmicb.2026.1850863 (PMC13534135; doi:10.3389/fmicb.2026.1850863)
Supplement: Supplementary file 1 [file Data_sheet_1.DOCX]

Supplementary Material

# 1 Supplementary Data

# 1.1 Supplementary Table S1

**Table 1:** Primer designing for gene expression

| **Gene** | **Primer** | **Sequence** | **Length** | **Tm**°C | **GC %** |
| --- | --- | --- | --- | --- | --- |
| ***PR-1*** | **Forward** | 5'ACACTTATGCCACCAATTCCT 3' | 21bases | 60.15 | 42 |
|  | **Reverse** | 3'GTAATGAACACATCTCCATCG5' | 21bases | 58.70 | 40 |
| ***UBQ*** | **Forward** | 5'GAAGGAGTCAACTCTCCACTTG3' | 22bases | 60.79 | 50 |
|  | **Reverse** | 3'ACTCTCCACCTCAAGGGTAATA5' | 22bases | 60.20 | 45 |

# 1.2 Supplementary Table S2

**Table 2:** **Morphological, biochemical and Plant growth promoting characteristics for identification of *B. subtilis* Medicinal 04 strain**

| **Test** | **Result** |
| --- | --- |
| **Morphological tests** | |
| Grams staining | + |
| Shape | Bacilli/Rod |
| Motility | + |
| Spore formation | + |
| **Biochemical tests** | |
| Indole production | - |
| Citrate utilization | + |
| Methyl red | - |
| Voges- Proskauer | + |
| Oxidase | - |
| Mannitol fermentation | + |
| Starch hydrolysis | + |
| Nitrate reduction | - |
| Casein hydrolysis | + |
| Gas production from glucose | - |
| **Plant growth promoting characteristics** | |
| IAA production | + |
| Phosphate solubilization | - |
| Ammonia production | + |
| Catalase production | + |
| Chitinolytic activity | ++ |

# 1.3 Supplementary Table S3

**Table 3: Morphological and physiological parameters of mung bean varieties (NM-51 and NM-20-21) across different treatments.**

| **Treatment** | **Shoot Length (cm)**  **(Mean**±SE) | | **Root Length (cm)**  **(Mean**±SE) | | **Number of Pods**  **(Mean**±SE) | | **Chlorophyll content**  **(Mean**±SE) | |
| --- | --- | --- | --- | --- | --- | --- | --- | --- |
|  | **NM-51** | **NM 20-21** | **NM-51** | **NM 20-21** | **NM-51** | **NM 20-21** | **NM-51** | **NM 20-21** |
| **HP** | 34.2±0.4 | 32.0 ±0.41 | 16.5±0.56 | 14.4±0.14 | 12.7±0.66 | 7.0±1.16 | 52.0±1.20 | 50.1±0.138 |
| **HP+F** | 32.4±0.29 | 30.8±0.31 | 15.5±0.40 | 14.0±0.08 | 13.7±0.33 | 7.67±0.33 | 48.7±0.88 | 46.3±1.42 |
| **HP+F +CC** | 30.7±0.33 | 28.7±0.66 | 14.4±0.30 | 14.0±0.52 | 8.3±0.66 | 6.0±0.57 | 41.7±0.88 | 35.3±1.35 |
| **HP+CC** | 29.2±0.59 | 26.3 ±0.56 | 13.7±0.24 | 12.1±0.18 | 7.0±0.57 | 5.0±0.57 | 40.6±1.18 | 34.0±0.32 |
| **BS (P)** | 35.8±0.31 | 34.4 ±0.43 | 18.4±0.24 | 16.2±0.37 | 14.0±0.57 | 8.6±0.33 | 54.2±0.35 | 53.4±0.45 |
| **CC +BS(P)** | 31.5 ±0.28 | 30.5 ±0.42 | 15.3±0.20 | 15.1±0.65 | 12.3±0.33 | 7.0±0.57 | 50.3±0.32 | 50.1±0.23 |
| **HP+BS (F)** | 34.7±0.66 | 32.5±0.37 | 16.6±0.44 | 14.7±0.38 | 12.7±0.33 | 8.3±0.66 | 53.4±0.28 | 53.5±0.22 |
| **HP+ BS(F)+CC** | 33.3±0.32 | 31.2 ±0.43 | 16.3±0.18 | 16.2±0.17 | 11.7±0.88 | 8.0±0.57 | 52.8±0.27 | 51.4±0.82 |

# 1.4 Supplementary Table S4

**Table 4: Biochemical and *PR-I e*xpression analysis across all treatments.**

| **Treatment** | **SOD (U/mg Protein)** | | **POD (U/mg Protein)** | | **CAT(U/mg Protein)** | | **PR-1 gene (Fold change)** | |
| --- | --- | --- | --- | --- | --- | --- | --- | --- |
|  | **NM-51**  **(Mean**±SE) | **NM 20-21**  **(Mean**±SE) | **NM-51**  **(Mean**±SE) | **NM 20-21**  **(Mean**±SE) | **NM-51**  **(Mean**±SE) | **NM 20-21**  **(Mean**±SE) | **NM-51**  **(Mean**±SE) | **NM 20-21**  **(Mean**±SE) |
| **HP** | 9.59±0.25 | 6.39±0.20 | 0.25±0.02 | 0.24±0.02 | 0.56±0.02 | 0.46±0.02 | 1.00±000 | 1.00±000 |
| **HP+F** | 9.34±0.18 | 6.34±0.003 | 0.22±0.01 | 0.24±0.003 | 0.56±0.004 | 0.49±0.0001 | 1.40±0.06 | 1.47±0.03 |
| **HP+F +CC** | 13.56±0.03 | 13.7±0.03 | 1.04±0.03 | 0.85±0.02 | 3.18±0.01 | 1.96±0.02 | 2.20±0.06 | 2.17±0.03 |
| **HP+CC** | 14.4±0.24 | 14.58±0.20 | 0.93±0.02 | 0.83±0.03 | 3.16±0.06 | 1.86±0.03 | 1.93±0.03 | 2.33±0.06 |
| **BS (P)** | 12.6±0.13 | 11.6±0.27 | 0.47±0.02 | 0.35±0.03 | 0.75±0.02 | 0.75±0.03 | 1.47±0.03 | 1.63±0.03 |
| **CC +BS(P)** | 16.33±0.21 | 20.61±0.25 | 1.37±0.04 | 1.24±0.02 | 3.94±0.02 | 2.39±0.05 | 2.57±0.03 | 2.73±0.07 |
| **HP+BS (F)** | 11.33±0.21 | 12.77±0.41 | 0.35±0.01 | 0.26±0.02 | 0.66±0.02 | 0.67±0.03 | 1.37±0.03 | 1.53±0.03 |
| **HP+ BS(F)+CC** | 14.65±0.29 | 16.61±0.25 | 1.18±0.05 | 1.10±0.05 | 2.55±0.03 | 1.96±0.02 | 2.67±0.03 | 2.83±0.07 |

**2.0 Supplementary Figures**

**Figure S1: Gene clusters encoding secondary metabolites in *B. subtilis* subsp. Subtilis str. 168 using antiSMASH**

**Figure S2: Gene clusters encoding secondary metabolites in *B. subtilis* Medicinal 04 using antiSMASH**
